# Supplementary material for: Conflict-climate-displacement: a cross-sectional ecological study determining the burden, risk and need for strategies for neglected tropical disease programmes in Africa
Source: BMJ Open. 2023 May 17;13(5):e071557. doi: 10.1136/bmjopen-2023-071557 (PMC10193097; doi:10.1136/bmjopen-2023-071557)
Supplement: Author's manuscript [file bmjopen-2023-071557.draft_revisions.pdf]

BMJ Open is committed to open peer review. As part of this commitment we make the peer review history of every article we publish publicly available.

When an article is published we post the peer reviewers' comments and the authors' responses online. We also post the versions of the paper that were used during peer review. These are the versions that the peer review comments apply to.

The versions of the paper that follow are the versions that were submitted during the peer review process. They are not the versions of record or the final published versions. They should not be cited or distributed as the published version of this manuscript.

BMJ Open is an open access journal and the full, final, typeset and author-corrected version of record of the manuscript is available on our site with no access controls, subscription charges or pay-per-view fees (http://bmjopen.bmj.com).

If you have any questions on BMJ Open's open peer review process please email info.bmjopen@bmj.com

BMJ Open

Conflict-climate-displacement: determining the burden, risk and need for strategies for Neglected Tropical Disease programmes in Africa.

Journal:	*BMJ Open*
Manuscript ID	bmjopen-2023-071557
Article Type:	Original research
Date Submitted by the Author:	02-Jan-2023
Complete List of Authors:	Kelly-Hope, Louise; University of Liverpool<br>Harding-Esch, Emma; London School of Hygiene and Tropical Medicine Department of Clinical Research<br>Willems, Johan; Christoffel-Blindenmission Christian Blind Mission (CBM)<br>Ahmed, Fatima; The MENTOR Initiative<br>Sanders, Angelia; The Carter Center
Keywords:	Public health < INFECTIOUS DISEASES, Epidemiology < TROPICAL MEDICINE, Epidemiology < INFECTIOUS DISEASES

SCHOLARONE™
Manuscripts

I, the Submitting Author has the right to grant and does grant on behalf of all authors of the Work (as defined in the below author licence), an exclusive licence and/or a non-exclusive licence for contributions from authors who are: i) UK Crown employees; ii) where BMJ has agreed a CC-BY licence shall apply, and/or iii) in accordance with the terms applicable for US Federal Government officers or employees acting as part of their official duties; on a worldwide, perpetual, irrevocable, royalty-free basis to BMJ Publishing Group Ltd ("BMJ") its licensees and where the relevant Journal is co-owned by BMJ to the co-owners of the Journal, to publish the Work in this journal and any other BMJ products and to exploit all rights, as set out in our licence.

The Submitting Author accepts and understands that any supply made under these terms is made by BMJ to the Submitting Author unless you are acting as an employee on behalf of your employer or a postgraduate student of an affiliated institution which is paying any applicable article publishing charge ("APC") for Open Access articles. Where the Submitting Author wishes to make the Work available on an Open Access basis (and intends to pay the relevant APC), the terms of reuse of such Open Access shall be governed by a Creative Commons licence – details of these licences and which Creative Commons licence will apply to this Work are set out in our licence referred to above.

Other than as permitted in any relevant BMJ Author's Self Archiving Policies, I confirm this Work has not been accepted for publication elsewhere, is not being considered for publication elsewhere and does not duplicate material already published. I confirm all authors consent to publication of this Work and authorise the granting of this licence.

For peer review only

Journal Article

TITLE: Conflict-climate-displacement: determining the burden, risk and need for strategies for Neglected Tropical Disease programmes in Africa.

AUTHORS: Louise A. Kelly-Hope[1*], Emma Harding-Esch[2], Johan Willems[3], Fatima Ahmed[4], Angelia M. Sanders[5]

* Corresponding author: L.Kelly-Hope@liverpool.ac.uk

AFFILIATIONS: *Note; Authors are members of the Neglected Tropical Diseases Non-Governmental Organisation Network (NNN), Conflict and Humanitarian Emergency (C&HE) Cross-Cutting Group - Mapping Task Team.*

1. Institute of Infection, Veterinary & Ecological Sciences, University of Liverpool, UK
2. London School of Hygiene & Tropical Medicine, London, UK.
3. CBM, Christoffel-Blindenmission Christian Blind Mission e.V., Bensheim, Germany.
4. The MENTOR Initiative, Haywards Heath, UK
5. The Carter Center, Atlanta, USA.

For peer review only

ABSTRACT

Objectives: Complex challenges such as political instability, climate change, and population displacement are increasing threats to national disease control, elimination, and eradication programmes. The objective of this study was to determine the burden and risk of conflict and climate related internal displacements and the need for strategies for countries endemic with Neglected Tropical Diseases (NTDs).

Design, setting and outcome measures: An ecological study was conducted including countries that are endemic with at least one of five NTDs requiring preventive chemotherapy in the African region. For each country, the number of NTDs, population size, and the number and rate per 100 000 of conflict-and natural disaster- internal displacements reported in 2021 were classified into high and low categories and used in unison to stratify and map the burden and risk.

Results: This analysis identified 45 NTD-endemic countries; eight countries were co-endemic with four or five diseases and had populations classified as “high” totalling more than 619 million people. We found 32 endemic countries had data on internal displacements related to conflict and disasters (n=16), disasters only (n=15) or conflict only (n=1). Six countries had both high conflict and disaster internal displacement numbers totalling more than 10.8 million people, and five countries had combined high conflict and disaster internal displacement rates, ranging from 770.8 to 7088.1 per 100 000 population. Weather-related hazards were the main cause of natural disaster displacements, predominately floods.

Conclusions: This paper presents a risk stratified approach to better understand the potential impact of these complex intersecting challenges. We advocate for a “call to action” to encourage national and international stakeholders to further develop, implement, and evaluate strategies to better assess NTD endemicity, and deliver interventions, in areas at risk of, or experiencing, conflict and climate disasters, in order to help meet the national targets.

For peer review only - http://bmjopen.bmj.com/site/about/guidelines.xhtml

STRENGTHS AND LIMITATIONS OF THIS STUDY

This study is the first to document the number of conflict- and natural disaster- related internal displacements in high Neglected Tropical Disease (NTDs) endemic countries in Africa.

The methodology employed is based on publicly available resources downloaded from the Expanded Special Project for Elimination of NTDs (ESPEN) data portal and the Internal Displacement Monitoring Centre (IDMC)

A new practical risk stratification approach is presented to help determine the intersecting challenges of NTDs and conflict and climate related internal displacement and highlights each country's unique combination of risk or vulnerability.

This information can be used for advocacy purposes to develop and implement mitigation strategies to help direct resources and enable effective NTD control and elimination programmes.

Future work needs to consider a range of data sources and spatial-temporal patterns to help identify hotspots, and determine the long-term implications and strategies needed for NTD programme activities.

For peer review only

INTRODUCTION

The Neglected Tropical Disease (NTD) road map 2021-2030 sets out aspiring global targets to prevent, control, eliminate or eradicate 20 NTDs in more than 100 endemic countries.[1] The roadmap highlights how reaching these goals can contribute to attaining the Sustainable Development Goals (SDGs),[2,3] and emphasises the need to accelerate programmatic action, intensify cross-cutting approaches, and change operating models and culture to facilitate country ownership. Importantly, it raises the need to address complex challenges such as climate change, political instability, and population movement and displacement, which threatens achieving the road map targets, and are increasing and intrinsically interlinked but have received little attention to-date.

Climate change can alter local ecology and disease dynamics.[4–6] It is expected to increase the intensity and frequency of extreme weather events and induce natural disasters such as floods and drought, which can restrict access to health care, limit water and food systems, cause outbreaks of climate-sensitive diseases, and lead to forced migration and internal displacement as living areas become inhabitable and livelihoods are destroyed.[7,8] The African continent is extremely vulnerable to climate change.[9] However there is a major knowledge gap associated with its impact on NTD transmission and programme activities across highly endemic countries.

Political instability can disrupt governance and escalate internal conflict and violence, and therefore restrict safe access to health care and potentially cause forced migration and internal displacement as people seek refuge. In Africa, the number of conflict events has increased dramatically in recent years[10,11] and impeded the effectiveness of many NTD programmes.[12,13] In response, practical approaches have been developed to address key issues such as safe and effective disease mapping and intervention strategies,[14–16] however more support to implement these at scale, and better understand the intersect with climate and complex humanitarian emergencies leading to displacement and how it affects vulnerable populations is urgently needed.[17]

External and internal displacement are driven by multiple causes and pose multiple risks for national programmes.[18] They can result in the introduction or recrudescence of infectious diseases, and the shortage of essential services such as health care, water and sanitation in areas with large unplanned population fluxes. These factors make it difficult for programmes to implement and assess the impact of interventions such as mass drug administration (MDA), which is being widely distributed as preventive chemotherapy for a number of NTDs.[1,19] The lack of specific protocols for distributing MDA to internally displaced populations and little understanding of the inhibiting factors that programmes face exacerbates the problem.

For peer review only - http://bmjopen.bmj.com/site/about/guidelines.xhtml

If the NTD road map 2021-2030 global targets are to be achieved, these challenges that NTD programmes face need to be at the forefront of national plans and stakeholder funding in the next decade. Currently, there are no formal health policies or guidelines on how national programmes may begin to document the burden, assess the impact, and develop strategies to overcome these pervasive threats, nor (to the best of our knowledge), specific funding streams exist for operational research and policy development. This is of significant concern and needs prioritising given that these challenges will drastically hinder progress, especially in Africa where many large, highly NTD endemic countries have additional programme challenges and are lagging behind key programmatic activities.[1,20]

The frequency of these displacement-related events in many NTD endemic countries is escalating, highlighting the need for urgent attention. The Internal Displacement Monitoring Centre (IDMC) highlights that conflict, violence and natural disasters caused approximately 38 million (M) internal displacements worldwide in 2021 with 80% of conflict and violence triggered displacements in sub-Saharan Africa alone.[21] Weather-related natural disasters such as storms and floods accounted for the vast majority of the 23.7M natural disaster (94%) driven internal displacements worldwide, underlining the threat of climate change.

NTD programmes need to better understand the burden and the extent to which these are singular or multiple intersecting challenges (Figure 1) to help to develop bespoke strategies and direct resources appropriately. This is important as some countries will experience more conflict- than climate, others more climate- than conflict-, or a complex combination of both conflict-climate.

Figure 1 here

Figure 1. Intersecting challenges of conflict, climate, and related internal displacement

We conducted a cross-sectional study to better understand the burden of conflict-, violence- and natural disaster-driven internal displacements in NTD endemic countries, and the extent to which these are singular or multiple intersecting challenges, to provide a basis from which bespoke strategies could be developed and resource allocation could be appropriately directed. We focused on countries in the World Health Organization (WHO) African region that are endemic for at least one of the five NTDs requiring preventive chemotherapy as a main intervention: lymphatic filariasis, onchocerciasis, schistosomiasis, soil-transmitted helminths, and trachoma.

For peer review only - http://bmjopen.bmj.com/site/about/guidelines.xhtml

METHODS

Neglected Tropical Disease and population data sources, classifications, and stratification

The NTD and population data were obtained from the Expanded Special Project for Elimination of NTDs (ESPEN) data portal, which collates data from national programmes and stakeholders on the various activities related to the implementation of MDA and/or morbidity management and disability (MMDP).[22] For each country, we summarised i) the number of NTDs requiring preventive chemotherapy and ii) the estimated country population based on 2020 data.

We then classified the countries endemic with four or five NTDs as '*High NTD number'* to reflect the complexity of managing many diseases and programmes, while the other countries were classified as '*Mod(erate)-Low NTD number*.' Second, we classified the ten countries with the highest populations (as an arbitrary threshold) as *'High population'* and others as '*Mod-Low population'*. Third, we combined the various classifications to develop four distinct categories, as shown here, and allocated them accordingly to each country.

NTD-population categories

- *High NTD number + High population*
- *High NTD number + Mod-Low population*
- *Mod-Low NTD number + High population*
- *Mod-Low NTD number + Mod-Low population*

Internal displacement data sources, classifications, and stratification

The data on internal displacements were obtained from the IDMC data portal, which monitors and collates country-specific data on internal displacements associated with conflict and generalised violence, and natural disasters (weather-related and geophysical) from a variety of sources.[21] It defines conflict displacements as the total number of internal displacements reported as a result of conflict and violence over the reporting year, and defines natural disaster displacements as the total number of internal displacements reported as a result of natural disasters over the reporting year. For this paper focusing on the concept of climate, the related internal displacements are referred to as natural disasters internal displacements as defined by IDMC and only include countries with reported data.

For each country, we summarised the number of conflict-and natural disaster- displacements based on 2021 reported data[21] and calculated crude rates of displacement per 100,000 population based on ESPEN population data.[22] Countries with no conflict and climate data for were not included in the analysis.

For peer review only - http://bmjopen.bmj.com/site/about/guidelines.xhtml

We then classified the 10 countries with the highest number and the highest rates of conflict-or disaster displaced populations as '*High conflict*' or '*High disaster*' and others as '*Mod-Low conflict*' or '*Mod-Low disaster*'. Second, the conflict and natural disaster classifications were then combined to develop four distinct categories each for numbers and rates as shown here and allocated to each country.

Displacement numbers and rate categories

- *High conflict + High disaster*
- *High conflict + Mod-Low disaster*
- *Mod-Low conflict + High disaster*
- *Mod-Low conflict + Mod-Low disaster*

Combined categories

All NTD, population and displacement categories were then combined to determine the overall patterns, and to help identify countries with high risk or vulnerability. For this paper, we highlighted selected high-risk combinations, which included the countries with a *High NTD number + High population* and a *High NTD number + Mod-Low population.*

Drivers of natural disaster internal displacement

To better understand the drivers of natural disaster internal displacement, we summarised the available IDMC data on hazard types in the countries with the highest natural disaster displacement numbers and rates in 2021. Hazard types were categorised as climate or weather-related (drought, flood, extreme temperature, storm, wet mass movement, and wildfire) and geophysical (earthquake, volcanic eruption, dry mass movement). The equivalent data for conflict internal displacement in 2021 were not available from this source and therefore not included in this paper.

Mapping

Maps for all classifications and stratifications were created using mapping software ArcGIS 10.8.1 (ESRI, Redland CA) and national administrative boundaries from the ESPEN data portal.[22]

Data limitations

We acknowledge that the use of open access data sources may have limitations if they are incomplete, and that our focus on a few selected data sources may cause unforeseen biases.

Patient and public involvement

No patient and/or members of the public were included in this study.

For peer review only - http://bmjopen.bmj.com/site/about/guidelines.xhtml

RESULTS

In total, 45 counties in the WHO African region had data on NTDs and populations (excluding Mauritius, Seychelles), and 32 countries reported data on conflict and/or natural disaster internal displacements; 16 countries reported both conflict and natural disaster; 15 countries reported natural disaster and one country reported conflict only internal displacement for 2021.

Neglected Tropical Diseases and population at risk

The total population in the 45 countries with data on NTDs was 1.1 billion. Thirty-one countries had a high number of NTDs: 12 countries endemic with five NTDs and 19 countries endemic with four NTDs (Figure 2A). The 10 countries with the highest populations are shown in Figure 2B, and of these, eight had both *High NTD number + High population* (Figure 2C) including (in descending order): Nigeria (est. 197.4M), Democratic Republic of Congo (DRC; 105.7M), Ethiopia (105.7M), Tanzania (mainland and Zanzibar - 55.9M), Kenya (52.0M), Uganda (41.0M), Angola (31.6M), Mozambique (29.9M) – totalling more than 619M people (Table 1).

Twelve countries had *Mod-Low NTD number + Mod-Low population* (Botswana, Cape Verde, Comoros, Eritrea, Eswatini, Lesotho, Mauritania, Namibia, Rwanda, Sao Tome and Principe, The Gambia, Togo), while 24 countries had a combination of *High NTD number + Mod-Low population* and one country (South Africa) had a *Mod-Low NTD number + High population* (Figure 2C).

Figure 2 here

Figure 2. Summary maps of Neglected Tropical Diseases, populations, and internal displacement numbers, rates classifications and risk combinations

Conflict and natural disaster internal displacement numbers and rates

Numbers: The total number of internal displacements in 2021 in the 31 high NTD endemic countries was 2.2M. The conflict displacement classifications for each country are shown in Figure 2D; the *High conflict* displacement numbers ranged from 0.1M-5.1M across 10 countries: Ethiopia (5.14M), DRC (2.71M), Burkina Faso (0.68M), Central African Republic (CAR; 0.50M), South Sudan (0.43M), Mali (0.25M), Nigeria (0.38M), Mozambique (0.19M), Cameroon (0.13M) and Niger (0.11M); the *Mod-Low Conflict* numbers across seven countries ranged from 0.94M – 0.04M.

The natural disaster displacement classifications are shown in Figure 2E; the *High Disaster* numbers ranged from 0.24M-0.89M across 10 countries, reported in DRC (0.89M), South Sudan (0.51M),

For peer review only - http://bmjopen.bmj.com/site/about/guidelines.xhtml

Ethiopia (0.24M), Niger (0.12M), Burundi (0.09M), Tanzania (0.05M), Uganda (0.05M), Mozambique (0.04M), Kenya (0.04M), Nigeria (0.02M); the *Mod-Low disaster* numbers in 20 countries ranged from 255 – 0.02M.

Six countries had both *High conflict + High disaster* internal displacement numbers, including (in descending order) Ethiopia (5.38M combined), DRC (3.60M), South Sudan (0.93M), Nigeria (0.40M), Mozambique (0.23M) and Niger (0.23M) (Figure 2F) – totalling more than 10.8M conflict and natural disaster combined internal displacements in 2021 (Table 1).

Table 1 here

Rates: The conflict internal displacement classifications are shown in Figure 2G; the *High conflict* displacement rates (Table 1) ranged from 252.7 – 9020.3 per 100,000 population, reported in CAR (9020.3 per 100,000), Ethiopia (4867.1), South Sudan (3255.6), Burkina Faso (3176.4), DRC (2564.7), Mali (1187.2), Mozambique (625.3), Cameroon (504.0), Niger (475.1), Chad (252.7); the *Mod-Low conflict* displacement rates ranged from 0.082 - 190.2 per 100,000.

The natural disaster internal displacement classifications are shown in Figure 2H; the *High disaster* displacement rates (Table 1) ranged from 144.1-3832.6 per 100,000 population, reported in South Sudan (3832.6 per 100,000), DRC (839.8), Burundi (754.7), Niger (507.6), CAR (429.3), Sao Tome and Principe (237.8), Ethiopia (227.2), Namibia (165.0), Mozambique (145.5), Chad (144.1); the *Mod-Low disaster* displacement rates ranged from 3.2 - 121.4 per 100,000.

Seven countries had both *High conflict + High disaster* internal displacement rates, including (in descending order) CAR (9449.7 combined), South Sudan (7088.1), Ethiopia (5094.3), DRC (3404.5), Niger (982.7), Mozambique (770.8) and Chad (398.8) (Figure 2I).

For peer review only

For peer review only - http://bmjopen.bmj.com/site/about/guidelines.xhtml

Table 1. **Summary of Neglected Tropical Diseases, populations, and internal displacement data and high-risk combinations in selected countries**

**Location**	**Number (No.) of NTDs and population***		**Internal displacement population numbers†**			**Internal displacement rates per 100,000 population**			**Combined classifications**
**Country**	**NTD No.**	**Population**	**Conflict No.**	**Natural Disaster No.**	**Conflict Disaster No.**	**Conflict rate**	**Natural Disaster rate**	**Conflict Disaster rate**	**NTD, Population Conflict, Natural Disaster**
**High NTDs + High population**									
DRC	5	105.7M	2,711,783	888,000	3.60M	2,564.7	839.8	3,404.5	High NTD-population+ conflict-disaster
Ethiopia	5	105.7M	5,142,356	240,009	5.38M	4867.1	227.2	5,094.3	High NTD-population+ conflict-disaster
Mozambique	4	29.9M	187,057	43,512	0.23M	625.3	145.5	770.8	High NTD-population+ conflict-disaster
Nigeria	5	197.4M	375,552	24,366	0.40M	190.2	12.3	202.6	
Tanzania	5	55.9M	0	46,707	0.05M	0	85.6	85.6	
Kenya	4	52.0M	4,634	36,442	0.04M	8.9	70.1	79.0	
Uganda	4	41.0M	1,278	46,690	0.05M	3.1	113.8	116.9	
Angola	4	31.6M	0	21727	0.02M	0	68.8	68.8	
**Summary**	**4-5**	**619M+**	**8,422,660**	**1,347,453**	**9.77M**	**0-4,867.1**	**12.3-839.8**	**68.8 – 5,094.3**	
**High NTD number + Mod-Low population**									
South Sudan	5	13.2M	429,432	505,544	0.93M	3255.6	3,832.6	7,088.1	High NTD-conflict-disaster
Niger	4	23.2M	110,203	117,747	0.23M	475.1	507.6	982.7	High NTD-conflict-disaster
Burkina Faso	4	21.5M	682,245	0	0.68M	3176.4	0	3176.4	High NTD - conflict
Cameroon	5	26.1M	131,452	1,764	0.13M	504.0	6.8	510.8	High NTD– conflict
CAR	5	5.5M	495,600	23,589	0.52M	9,020.3	429.3	9,449.7	High NTD - conflict
Mali	4	21.0M	249,174	5,994	0.26M	1,187.2	28.6	1,215.7	High NTD - conflict
Burundi	4	11.5M	94	86,885	0.09M	0.8	754.7	755.5	High NTD - disaster
Chad	5	16.8M	42,355	24,144	0.07M	252.7	144.1	398.8	
**Summary**	**4-5**	**138.8M**	**2,140,555**	**765,667**	**2.91M**	**0.8-9,020.3**	**0 – 3,832.6**	**398.8 – 9,449.7**	

*ESPEN data 2021[22]
† IDMC data 2019-2021[21]
Shading represents countries with a classified High NTD number, High population, or *High Conflict* and/or *High Natural disaster* internal displacement numbers and/or rates

For peer review only - http://bmjopen.bmj.com/site/about/guidelines.xhtml

Risk combinations

Of the countries with *High NTD number + High population*, we found that DRC, Ethiopia, Mozambique, and Nigeria had *High conflict + High disaster* displacement numbers (Figure 2J), and DRC, Ethiopia, and Mozambique had *High conflict + High disaster* displacement rates (Figure 2K) in 2021. Collectively, these three countries reported more than 241M people at risk of four to five NTDs and 9.2M conflict and/or natural disaster internal displacements in 2021 alone; these may be considered the most at risk or vulnerable countries in the African region. Nigeria was found to have *High conflict + High disaster* displacement numbers, while Tanzania, Kenya, and Uganda had among the highest *High disaster* displacement numbers (Table 1).

Of the countries with high NTD numbers and displacement, we found South Sudan and Niger to have both *High conflict + High disaster* number and rates, while Burkina Faso, Cameroon, CAR, and Mali had both *High conflict* numbers and rates, and Burundi had both *High disaster* displacement numbers and rates in 2021 (Table 1).

Drivers of natural disaster internal displacement

The number of reported natural disaster hazard events in 2021 varied across countries, with the highest frequency reported in Burundi (n=80), Uganda (n=60) and South Sudan (n=31), and the highest number of hazard-related internal displacements reported in DRC (0.89M), South Sudan (0.51M), and Ethiopia (0.24M) (Table 2). In all countries, weather-related hazards were most common with floods the dominant hazard type, accounting for around half (55.2%) of all natural disaster displacements across 12 countries, except DRC where a volcanic disruption led to 0.6M displacements, and in Mozambique where a storm led to 0.04M displacements. In total, 150 flood events across 11 countries caused 0.85M natural disaster hazard related internal displacements. Other causes of internal displacement included mass wet or dry movement and wildfires but affected far fewer numbers.

For peer review only - http://bmjopen.bmj.com/site/about/guidelines.xhtml

Table 2. Summary of natural disaster internal displacements by hazard type in selected countries

Country	Total Population.	Total number (No.) of Natural Disaster Displacements	Total rate of Natural Disaster Displacements	Total No. hazard events	No. of natural disaster displacements by hazard type (No. of hazard events)			
					Flood	Drought	Storm	Other*
**High NTD number, High population, High climate numbers or rates**								
DRC	105.7M	0.89M	2564.7	21	0.26M (n=10)		0.002M (n=8)	0.6M (n=3)
Ethiopia	105.7M	0.24M	839.8	7	0.19M (n=4)	0.05M (n=1)		0.00001 (n=2)
Mozambique	29.9M	0.04M	145.5	2			0.04M (n=2)	
Nigeria	197.4M	0.02M	12.3	31	0.02M (n=31)			
Tanzania	55.0M	0.05M	85.6	6	0.02M (n=2)		0.03M (n=4)	
Kenya	52.0M	0.04M	70.1	3	0.04M (n=2)		0.000M (n-1)	
Uganda	41.0M	0.05M	113.8	60	0.04M (n=24)		0.005M (n=28)	0.001M (n=8)
**Summary**	**586.7M**	**1.33**	**12.3 – 2564.7**	**130**	**0.57M (n=73)**	**0.05M (n=1)**	**0.08 (n=43)**	**0.60 (n=13)**
**High NTD number, Mod-Low population, high natural disaster numbers or rates**								
South Sudan	13.2M	0.05M	3832.6	39	0.05M (n=39)			
Niger	23.2M	0.12M	507.6	1	0.12M (n=1)			
CAR	5.5M	0.02M	429.3	16	0.02 (n=16)			
Burundi	11.5M	0.26M	754.7	80	0.07M (n=15)		0.013M (n=63)	0.0002 (n=1)
Chad	16.8M	0.02M	144.1	6	0.02 (n=6)			
**Summary**	**70.2M**	**0.47M**	**144.1 – 3832.6**	**142**	**0.28 (n=77)**		**0.013M (n=63)**	**0.0002 (n=1)**

Data source iDMC (numbers rounded)[21]
*Other category included – wet or dry mass movement and/or wildfires

For peer review only

DISCUSSION

This paper highlights that many African countries have high numbers of NTDs, which require a great deal of coordination and logistics in-country to implement routine NTD programming.[22] In the eight most populous endemic countries, we quantified that more than half a billion people will need multiple NTD interventions and long-term surveillance activities in the next decade. Routine NTD programming has been and will be negatively impacted by large population displacements, which adds to the existing difficulties of determining accurate population numbers in countries where there is a lack of regular census data.[23,24] The ESPEN data portal provides population estimates[22] and could start to collate data on internal displacements from various sources, including NTD programme managers who are experiencing the challenges first hand and may provide more detailed information and invaluable insights into these problems.

Alarmingly, here we show in just one year that Ethiopia, DRC, South Sudan, Nigeria, Mozambique, and Niger reported more than 10M internal conflict and natural disaster displacements and have among them the highest conflict and natural disaster rates per population. If current trends continue or increase, these countries are at high risk of not reaching the NTD road map targets and will require significant technical and financial support from stakeholders if global goals are to be met.[1] This is critical as these six countries have among the lowest levels of human development in the world,[25] and may not be able to access and mobilise in-country resources needed to accelerate programmatic action, intensify cross-cutting approaches, and change operating models to facilitate enhanced country ownership as set out in the NTD road map 2021-2030.[1]

We limited our analysis to one internal displacement data source but advocate that future research should aim to triangulate information from other data sources to build a comprehensive picture of the situation. A further limitation was that we applied simple classifications and risk combinations to the NTD, population, and internal displacement figures to allow us to initially focus on countries most affected as a means of understanding the magnitude of these complex challenges. This does not imply that countries with lower numbers or rates of NTDs, population, conflict and natural disaster internal displacements are less of a priority for NTD programmes, but rather the magnitude is different. All endemic countries in Africa and elsewhere will need support to address these challenges and future work should consider more advanced analysis applied at a subnational level and include additional compounding factors such as health infrastructure, community accessibility, and strength of the NTD programme.[12,18]

For peer review only - http://bmjopen.bmj.com/site/about/guidelines.xhtml

Further, future work also needs to consider the spatial-temporal patterns and geographical overlap of conflict and natural disaster driven displacement to identify hotspots, and determine the long-term implications and strategies needed for NTD programme activities. For example, if there is protracted conflict in an area, then programmes can adapt their methods of programmatic delivery, target non-conflict areas, or identify methods of treating displaced populations that have sought refuge in secure areas.[15] If flooding is increasingly predictable in an area, then programmes can adapt the timing of programme activities in that area or identify alternate treatment sites for those displaced by the flooding. Lesson learned from other regions

The African continent is predicted to continue experiencing negative effects from climate change due to increasing temperatures, rising sea-levels, cumulative failed rainy seasons, drought, and shrinking surface water.[8] These events and resulting food and water scarcity can feed into political instability, and exacerbate ongoing tensions thereby creating a vulnerability multiplier for those at risk of conflict, natural disasters, and NTDs. Climate change is an escalating security threat,[26–29] and an understanding of the key drivers is critical in Africa and elsewhere. Here we highlight that most natural disaster hazards were climate or weather-related events, especially flooding which led to nearly one million displacements in 11 African countries in one year alone. However, the impact of these events on disease transmission dynamics in highly endemic areas, and how they affect programmes, is largely unknown as very little research has been conducted in Africa and elsewhere.[4–6]

Recommendations

We advocate for a "call to action" to encourage policy makers, programme managers, researchers, implementing partners and donors to develop, implement, and evaluate a set of strategies to better assess NTD burden in areas at risk or experiencing conflict and climate related natural disasters, and deliver interventions that are urgently required. Here we focus on African countries, but the same methodologies may be applied to different WHO regions to understand the distinct challenges. There is a need to build on core principles already being developed by experienced teams that may address these special populations and problems.[14,30] As a first step, we suggest an overarching set of recommendations that include the development of preferred practices; data platform; decision support tool, advocacy and communication and implementation research strategies as outlined in Box 1.

For peer review only - http://bmjopen.bmj.com/site/about/guidelines.xhtml
For peer review only

Box 1. Overarching recommendations to address complex challenges in NTD endemic countries

- PREFERRED PRACTICES: A set of informed preferred practice documents outlining potential step-by step processes and methods to address singular or multiple intersecting conflict- climate-displacement challenges that impede NTD programmatic activities.
- DATA PLATFORM: A central data portal that combines data for each country on: NTD burden, population size, internal and cross-border displacement numbers (by conflict, natural disaster, and hazard type categorisation) and administrative boundaries.
- DECISION SUPPORT TOOL: A practical online tool that facilitates the national NTD programme teams to input and analysis conflict-climate-displacement data to assess risk and support programmatic decision-making at implementation unit or district level.
- ADVOCACY AND COMMUNICATION. An advocacy and communication plan with goals to document and inform national and international stakeholders of the key threats and success stories related to the conflict-climate-displacement challenges that NTD programmes confront.
- IMPLEMENTATION RESEARCH: A strategic research agenda outlining the range of conflict- climate-displacement challenges, and the potential research questions, methodological approaches, and funding opportunities that could be drawn on by NTD programmes and academic partners.

CONCLUSION

This paper highlights the urgent need for health policies and guidelines to address the escalating risks of conflict, climate change-induced natural disasters, and associated internal displacement of populations in NTD endemic countries in Africa. We have shown that publicly available data sources could potentially be used by national policy makers and NTD programmes as a first step to better understanding the intersecting challenges and their level of risk and vulnerability. This would allow them to develop risk assessments and mitigation strategies that may be tailored to the local context and health needs of internally displaced persons, given that the types and burden of NTDs, and the causes of internal displacement, vary within and between countries. There is a need to build new stakeholder collaborations and a collective agenda for these neglected conflict-climate-displacement challenges, so that NTD programmes can meet the 2021-2030 road map targets and 2030 Agenda for Sustainable Development and ensure that no one is left behind.

For peer review only - http://bmjopen.bmj.com/site/about/guidelines.xhtml

CONTRIBUTORS

The conception for the paper was initiated by LAK-H, and further developed based on discussions and email exchanges with all authors who are members of the Neglected Tropical Diseases Non-Governmental Organisation Network (NNN), Conflict and Humanitarian Emergency (C&HE) Cross-Cutting Group - Mapping Task Team https://www.ntd-ngonetwork.org/cross-cutting-groups/conflict-and-humanitarian-emergencies . LAK-H wrote the first draft and all other authors contributed further comments and suggestions on subsequent drafts.

DATA AVAILABILITY STATEMENT

All data relevant to this study are included in the article or available in the public, open access repositories.

ETHICS STATEMENT

Patient consent for publication

This study does not involve human participants and ethical approval was not required.

COMPETING INTERESTS

None declared

ROLE OF THE FUNDER

No funding was required for this paper.

For peer review only

REFERENCES

1 World Health Organization. Ending the neglect to attain the sustainable development goals: a road map for neglected tropical diseases 2021–2030. Geneva: World Health Organization, 2020 https://apps.who.int/iris/handle/10665/338565 (accessed Aug 4, 2022).

2 Bangert M, Molyneux DH, Lindsay SW, Fitzpatrick C, Engels D. The cross-cutting contribution of the end of neglected tropical diseases to the sustainable development goals. *Infectious Diseases of Poverty* 2017; **6**: 73.

3 United Nations.Department of Economic and Social Affairs. Sustainable Development Goals. Sustainable Development. https://sdgs.un.org/goals (accessed Dec 14, 2022).

4 Tidman R, Abela-Ridder B, de Castañeda RR. The impact of climate change on neglected tropical diseases: a systematic review. *Transactions of The Royal Society of Tropical Medicine and Hygiene* 2021; **115**: 147–68.

5 Bryson JM, Bishop-Williams KE, Berrang-Ford L, *et al.* Neglected Tropical Diseases in the Context of Climate Change in East Africa: A Systematic Scoping Review. *The American Journal of Tropical Medicine and Hygiene* 2020; **102**: 1443–54.

6 Booth M. Chapter Three - Climate Change and the Neglected Tropical Diseases. In: Rollinson D, Stothard JR, eds. Advances in Parasitology. Academic Press, 2018: 39–126.

7 Eckstein D, Künzel V, Schäfer L. Global Climate Risk Index 2021 Who Suffers Most Extreme Weather Events? Weather-Related Loss Events in 2019 and 2000-2019. 2021.

8 World Meteorological Organization. State of Climate Africa 2021. 2022 https://library.wmo.int/index.php?lvl=notice_display&id=22125#.Y5l2B-zP2lM (accessed Dec 14, 2022).

9 Zielinski C. COP27 climate change conference: urgent action needed for Africa and the world. *BMJ Global Health* 2022; **7**: e010965.

10 Palik J, Rustad SA, Methi F. Conflict Trends in Africa, 1989–2019. .

11 Raleigh C, Linke A, Hegre H, Karlsen J. Introducing ACLED: An Armed Conflict Location and Event Dataset: Special Data Feature. *Journal of Peace Research* 2010; **47**: 651–60.

12 Harvey D, Shu'aibu J, Debam MT, Aba AK, Torres-Vitolas CA. How can the neglected tropical disease community be inclusive and equitable in programme delivery? Reaching refugees and internally displaced persons through integrating a 'leave no one behind' approach. *Int Health* 2022; **14**: ii33–7.

13 Yaya G. Lessons learned in the implementation of programmes to eliminate trachoma within conflict zones. *Trans R Soc Trop Med Hyg* 2022; : trac061.

14 Neglected Tropical Disease NGO Network (NNN). Conflict and Humanitarian Emergencies. https://www.ntd-ngonetwork.org/cross-cutting-groups/conflict-and-humanitarian-emergencies (accessed Aug 24, 2022).

For peer review only

15 Kelly-Hope LA, Sanders AM, Harding-Esch E, *et al.* Complex emergencies and the control and elimination of neglected tropical diseases in Africa: developing a practical approach for implementing safe and effective mapping and intervention strategies. *Confl Health* 2021; **15**: 18.

16 Kelly-Hope LA, Molyneux DH. Quantifying conflict zones as a challenge to certification of Guinea worm eradication in Africa: a new analytical approach. *BMJ Open* 2021; **11**: e049732.

17 Cantor D, Swartz J, Roberts B, *et al.* Understanding the health needs of internally displaced persons: A scoping review. *J Migr Health* 2021; **4**: 100071.

18 Adams MW, Sutherland EG, Eckert EL, Saalim K, Reithinger R. Leaving no one behind: targeting mobile and migrant populations with health interventions for disease elimination—a descriptive systematic review. *BMC Med* 2022; **20**: 172.

19 World Health Organization. Summary of global update on implementation of preventive chemotherapy against NTDs in 2020. 2021.

20 Bockarie MJ, Kelly-Hope LA, Rebollo M, Molyneux DH. Preventive chemotherapy as a strategy for elimination of neglected tropical parasitic diseases: endgame challenges. *Philos Trans R Soc Lond B Biol Sci* 2013; **368**: 20120144.

21 Internal Displacement Monitoring Centre (IDMC). Global Internal Displacement Database. Data (2022). https://www.internal-displacement.org/database/displacement-data.

22 World Health Organization. Expanded Special Project for Elimination of Neglected Tropical Diseases (ESPEN). 2022. https://espen.afro.who.int/.

23 Carlin, Alexandra. Why we should reconsider population data for disease control and public health programmes. BMC BugBitten. 2021; published online Nov 26. https://blogs.biomedcentral.com/bugbitten/2021/11/26/population-data-disease-control-public-health/ (accessed Oct 10, 2022).

24 Ratnayake R, Abdelmagid N, Dooley C. What we do know (and could know) about estimating population sizes of internally displaced people. *Journal of Migration and Health* 2022; **6**: 100120.

25 United Nations Develop Programme (UNDP). Human Development Report 2021-22: Uncertain Times, Unsettled Lives: Shaping our Future in a Transforming World. New York, 2022 https://hdr.undp.org/data-center/human-development-index#/indicies/HDI (accessed Oct 10, 2022).

26 United Nations Environment Programme (UNEP). Climate change and security risks. Disasters and conflicts. 2022. https://www.unep.org/explore-topics/disasters-conflicts/what-we-do/disaster-risk-reduction/climate-change-and-security (accessed Oct 13, 2022).

27 Brown O, Hammill A, Mcleman R. Climate change as the 'new' security threat: implications for Africa. *International Affairs* 2007; **83**: 1141–54.

28 Evans MS, Munslow B. Climate change, health, and conflict in Africa's arc of instability. *Perspect Public Health* 2021; **141**: 338–41.

29 Mach KJ, Kraan CM, Adger WN, *et al.* Climate as a risk factor for armed conflict. *Nature* 2019; **571**: 193–7.

For peer review only

For peer review only - http://bmjopen.bmj.com/site/about/guidelines.xhtml

30 The International, Coalition for Trachoma Control, (ICTC). THE END IN SIGHT: From collaboration to elimination, strategic plan 2022–2030. 2022; published online May. https://www.trachomacoalition.org/sites/default/files/content/resources/files/ICTC%20Strategic%20Plan.pdf (accessed Oct 10, 2022).

For peer review only

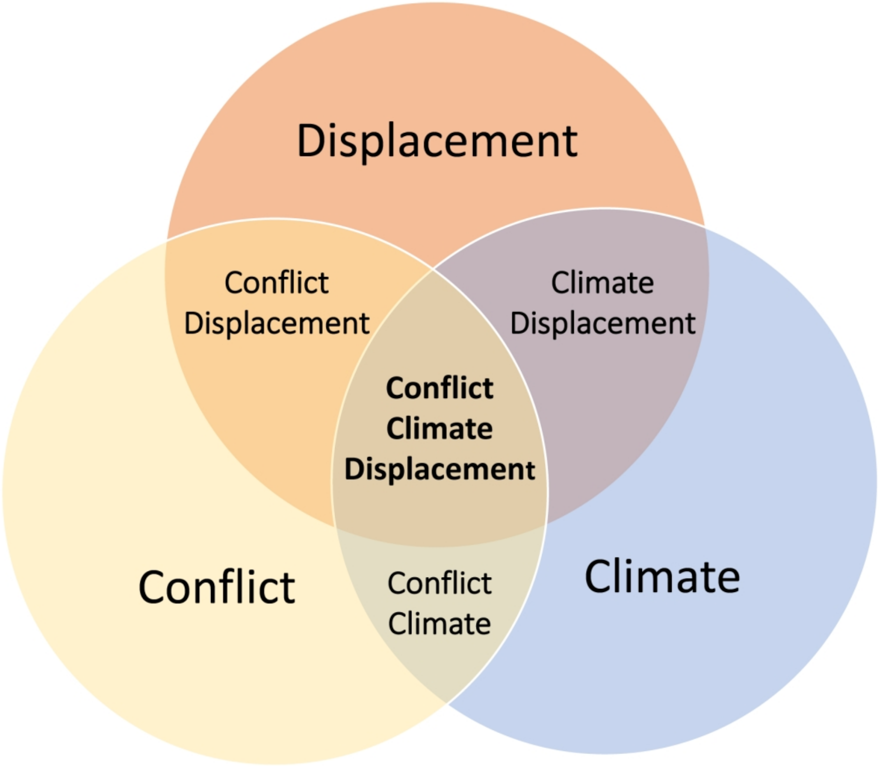

164x147mm (300 x 300 DPI)

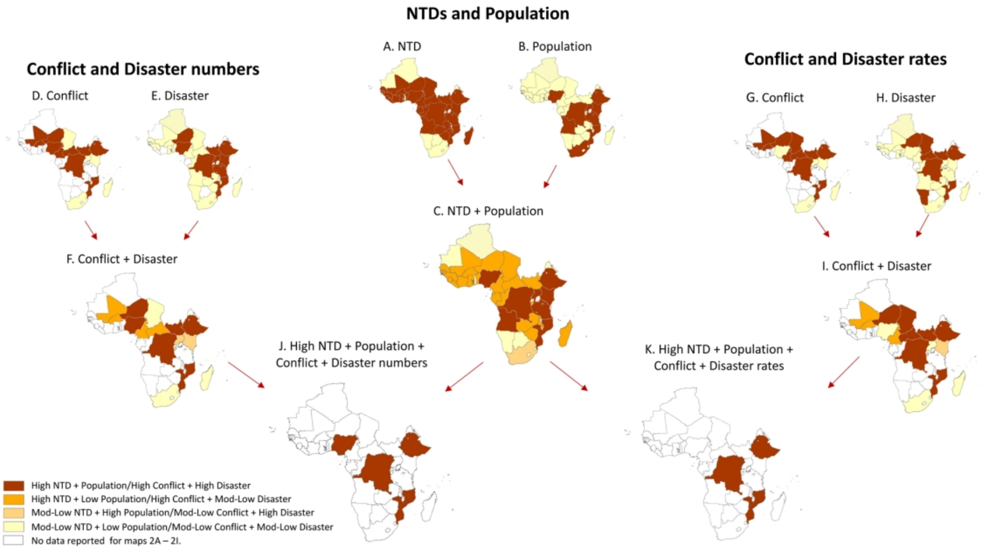

170x95mm (300 x 300 DPI)

STROBE Statement—Checklist of items that should be included in reports of ***cross-sectional studies***

	Item No	Recommendation	Page No
**Title and abstract**	1	(*a*) Indicate the study's design with a commonly used term in the title or the abstract	2
		(*b*) Provide in the abstract an informative and balanced summary of what was done and what was found	2
**2Introduction**			
Background/rationale	2	Explain the scientific background and rationale for the investigation being reported	4-5
Objectives	3	State specific objectives, including any prespecified hypotheses	5
**Methods**			
Study design	4	Present key elements of study design early in the paper	5
Setting	5	Describe the setting, locations, and relevant dates, including periods of recruitment, exposure, follow-up, and data collection	6
Participants	6	(*a*) Give the eligibility criteria, and the sources and methods of selection of participants	6-7
Variables	7	Clearly define all outcomes, exposures, predictors, potential confounders, and effect modifiers. Give diagnostic criteria, if applicable	6-7
Data sources/ measurement	8*	For each variable of interest, give sources of data and details of methods of assessment (measurement). Describe comparability of assessment methods if there is more than one group	*6-7*
Bias	9	Describe any efforts to address potential sources of bias	6
Study size	10	Explain how the study size was arrived at	6-7
Quantitative variables	11	Explain how quantitative variables were handled in the analyses. If applicable, describe which groupings were chosen and why	6-7
Statistical methods	12	(*a*) Describe all statistical methods, including those used to control for confounding	N/A
		(*b*) Describe any methods used to examine subgroups and interactions	6-7
		(*c*) Explain how missing data were addressed	6
		(*d*) If applicable, describe analytical methods taking account of sampling strategy	6-7
		(*e*) Describe any sensitivity analyses	N/A
**Results**			
Participants	13*	(a) Report numbers of individuals at each stage of study—eg numbers potentially eligible, examined for eligibility, confirmed eligible, included in the study, completing follow-up, and analysed	N/A
		(b) Give reasons for non-participation at each stage	N/A
		(c) Consider use of a flow diagram	Figure 2
Descriptive data	14*	(a) Give characteristics of study participants (eg demographic, clinical, social) and information on exposures and potential confounders	8-12
		(b) Indicate number of participants with missing data for each variable of interest	N/A
Outcome data	15*	Report numbers of outcome events or summary measures	8-12

For peer review only - http://bmjopen.bmj.com/site/about/guidelines.xhtml

Main results	16	(*a*) Give unadjusted estimates and, if applicable, confounder-adjusted estimates and their precision (eg, 95% confidence interval). Make clear which confounders were adjusted for and why they were included	N/A
		(*b*) Report category boundaries when continuous variables were categorized	8-12
		(*c*) If relevant, consider translating estimates of relative risk into absolute risk for a meaningful time period	N/A
Other analyses	17	Report other analyses done—eg analyses of subgroups and interactions, and sensitivity analyses	8-12
**Discussion**			
Key results	18	Summarise key results with reference to study objectives	13
Limitations	19	Discuss limitations of the study, taking into account sources of potential bias or imprecision. Discuss both direction and magnitude of any potential bias	13-14
Interpretation	20	Give a cautious overall interpretation of results considering objectives, limitations, multiplicity of analyses, results from similar studies, and other relevant evidence	13-14
Generalisability	21	Discuss the generalisability (external validity) of the study results	13-14
**Other information**			
Funding	22	Give the source of funding and the role of the funders for the present study and, if applicable, for the original study on which the present article is based	16

*Give information separately for exposed and unexposed groups.

Note: An Explanation and Elaboration article discusses each checklist item and gives methodological background and published examples of transparent reporting. The STROBE checklist is best used in conjunction with this article (freely available on the Web sites of PLoS Medicine at http://www.plosmedicine.org/, Annals of Internal Medicine at http://www.annals.org/, and Epidemiology at http://www.epidem.com/). Information on the STROBE Initiative is available at www.strobe-statement.org.

For peer review only

For peer review only - http://bmjopen.bmj.com/site/about/guidelines.xhtml

BMJ Open

Conflict-climate-displacement: determining the burden, risk and need for strategies for Neglected Tropical Disease programmes in Africa.

Journal:	*BMJ Open*
Manuscript ID	bmjopen-2023-071557.R1
Article Type:	Original research
Date Submitted by the Author:	22-Mar-2023
Complete List of Authors:	Kelly-Hope, Louise; University of Liverpool<br>Harding-Esch, Emma; London School of Hygiene & Tropical Medicine, Clinical Research Department<br>Willems, Johan; Christoffel-Blindenmission Christian Blind Mission (CBM)<br>Ahmed, Fatima; The MENTOR Initiative<br>Sanders, Angelia; The Carter Center
**Primary Subject Heading**:	Global health
Secondary Subject Heading:	Epidemiology, Health policy, Infectious diseases, Public health
Keywords:	Public health < INFECTIOUS DISEASES, Epidemiology < TROPICAL MEDICINE, Epidemiology < INFECTIOUS DISEASES, Neglected Diseases

SCHOLARONE™
Manuscripts

I, the Submitting Author has the right to grant and does grant on behalf of all authors of the Work (as defined in the below author licence), an exclusive licence and/or a non-exclusive licence for contributions from authors who are: i) UK Crown employees; ii) where BMJ has agreed a CC-BY licence shall apply, and/or iii) in accordance with the terms applicable for US Federal Government officers or employees acting as part of their official duties; on a worldwide, perpetual, irrevocable, royalty-free basis to BMJ Publishing Group Ltd ("BMJ") its licensees and where the relevant Journal is co-owned by BMJ to the co-owners of the Journal, to publish the Work in this journal and any other BMJ products and to exploit all rights, as set out in our licence.

The Submitting Author accepts and understands that any supply made under these terms is made by BMJ to the Submitting Author unless you are acting as an employee on behalf of your employer or a postgraduate student of an affiliated institution which is paying any applicable article publishing charge ("APC") for Open Access articles. Where the Submitting Author wishes to make the Work available on an Open Access basis (and intends to pay the relevant APC), the terms of reuse of such Open Access shall be governed by a Creative Commons licence – details of these licences and which Creative Commons licence will apply to this Work are set out in our licence referred to above.

Other than as permitted in any relevant BMJ Author's Self Archiving Policies, I confirm this Work has not been accepted for publication elsewhere, is not being considered for publication elsewhere and does not duplicate material already published. I confirm all authors consent to publication of this Work and authorise the granting of this licence.

For peer review only

Journal Article

TITLE: Conflict-climate-displacement: determining the burden, risk and need for strategies for Neglected Tropical Disease programmes in Africa.

AUTHORS: Louise A. Kelly-Hope[1*], Emma Harding-Esch[2], Johan Willems[3], Fatima Ahmed[4], Angelia M. Sanders[5]

* Corresponding author: lkhope@liverpool.ac.uk

AFFILIATIONS: *Note; Authors are members of the Neglected Tropical Diseases Non-Governmental Organisation Network (NNN), Conflict and Humanitarian Emergency (C&HE) Cross-Cutting Group - Mapping Task Team.*

1. Institute of Infection, Veterinary & Ecological Sciences, University of Liverpool, UK
2. London School of Hygiene & Tropical Medicine, London, UK.
3. CBM, Christoffel-Blindenmission Christian Blind Mission e.V., Bensheim, Germany.
4. The MENTOR Initiative, Haywards Heath, UK
5. The Carter Center, Atlanta, USA.

For peer review only - http://bmjopen.bmj.com/site/about/guidelines.xhtml

ABSTRACT

Objectives: Complex challenges such as political instability, climate change, and population displacement are increasing threats to national disease control, elimination, and eradication programmes. The objective of this study was to determine the burden and risk of conflict and climate related internal displacements and the need for strategies for countries endemic with Neglected Tropical Diseases (NTDs).

Design, setting and outcome measures: An ecological study was conducted including countries that are endemic with at least one of five NTDs requiring preventive chemotherapy in the African region. For each country, the number of NTDs, population size, and the number and rate per 100 000 of conflict-and natural disaster- internal displacements reported in 2021 were classified into high and low categories and used in unison to stratify and map the burden and risk.

Results: This analysis identified 45 NTD-endemic countries; eight countries were co-endemic with four or five diseases and had populations classified as "high" totalling more than 619 million people. We found 32 endemic countries had data on internal displacements related to conflict and disasters (n=16), disasters only (n=15) or conflict only (n=1). Six countries had both high conflict and disaster internal displacement numbers totalling more than 10.8 million people, and five countries had combined high conflict and disaster internal displacement rates, ranging from 770.8 to 7088.1 per 100 000 population. Weather-related hazards were the main cause of natural disaster displacements, predominately floods.

Conclusions: This paper presents a risk stratified approach to better understand the potential impact of these complex intersecting challenges. We advocate for a "call to action" to encourage national and international stakeholders to further develop, implement, and evaluate strategies to better assess NTD endemicity, and deliver interventions, in areas at risk of, or experiencing, conflict and climate disasters, in order to help meet the national targets.

For peer review only - http://bmjopen.bmj.com/site/about/guidelines.xhtml

STRENGTHS AND LIMITATIONS OF THIS STUDY

This study is the first to document the number of conflict- and natural disaster- related internal displacements in high Neglected Tropical Disease (NTDs) endemic countries in Africa.

The methodology employed is based on publicly available resources downloaded from the Expanded Special Project for Elimination of NTDs (ESPEN) data portal and the Internal Displacement Monitoring Centre (IDMC).

A new practical risk stratification approach is presented to help determine the intersecting challenges of NTDs and conflict- and natural disaster- related internal displacements, and highlights each country's unique combination of risk or vulnerability.

This information can be used for advocacy purposes to develop and implement mitigation strategies to help direct resources and enable effective NTD control and elimination programmes.

Future work needs to consider a range of data sources and spatial-temporal patterns to help identify hotspots, and determine the long-term implications and strategies needed for NTD programme activities.

For peer review only

INTRODUCTION

The Neglected Tropical Disease (NTD) road map 2021-2030 sets out aspiring global targets to prevent, control, eliminate or eradicate 20 NTDs in more than 100 endemic countries.[1] The roadmap highlights how reaching these goals can contribute to attaining the Sustainable Development Goals (SDGs),[2,3] and emphasises the need to accelerate programmatic action, intensify cross-cutting approaches, and change operating models and culture to facilitate country ownership. Importantly, it raises the need to address complex challenges such as climate change, political instability, and population movement and displacement, which threatens achieving the road map targets, and are increasing and intrinsically interlinked but have received little attention to-date.

Climate change can alter local ecology and disease dynamics.[4–6] It is expected to increase the intensity and frequency of extreme weather events and induce natural disasters such as floods and drought, which can restrict access to health care, limit water and food systems, cause outbreaks of climate-sensitive diseases, and lead to forced migration and internal displacement as living areas become inhabitable and livelihoods are destroyed.[7,8] The African continent is extremely vulnerable to climate change.[9] However there is a major knowledge gap associated with its impact on NTD transmission and programme activities across highly endemic countries.

Political instability can disrupt governance and escalate internal conflict and violence, and therefore restrict safe access to health care and potentially cause forced migration and internal displacement as people seek refuge. In Africa, the number of conflict events has increased dramatically in recent years[10,11] and impeded the effectiveness of many NTD programmes.[12–15] In response, practical approaches have been developed to address key issues such as safe and effective disease mapping and intervention strategies,[16–18] however more support to implement these at scale, and better understand the intersect with climate and complex humanitarian emergencies leading to displacement and how it affects vulnerable populations is urgently needed.[19]

External and internal displacement are driven by multiple causes and pose multiple risks for national programmes.[20] They can result in the introduction or recrudescence of infectious diseases, and the shortage of essential services such as health care, water and sanitation in areas with large unplanned population fluxes. These factors make it difficult for programmes to implement and assess the impact of interventions such as mass drug administration (MDA), which is being widely distributed as preventive chemotherapy for a number of NTDs.[1,21] The lack of specific protocols for distributing MDA to internally displaced populations and little understanding of the inhibiting factors that programmes face exacerbates the problem.

For peer review only - http://bmjopen.bmj.com/site/about/guidelines.xhtml

If the NTD road map 2021-2030 global targets are to be achieved, these challenges that NTD programmes face need to be at the forefront of national plans and stakeholder funding in the next decade. Currently, there are no formal health policies or guidelines on how national programmes may begin to document the burden, assess the impact, and develop strategies to overcome these pervasive threats, nor (to the best of our knowledge), specific funding streams exist for operational research and policy development. This is of significant concern and needs prioritising given that these challenges will drastically hinder progress, especially in Africa where many large, highly NTD endemic countries have additional programme challenges and are lagging behind key programmatic activities.[1,22]

The frequency of these displacement-related events in many NTD endemic countries is escalating, highlighting the need for urgent attention. The Internal Displacement Monitoring Centre (IDMC) highlights that conflict, violence and natural disasters caused approximately 38 million (M) internal displacements worldwide in 2021 with 80% of conflict and violence triggered displacements in sub-Saharan Africa alone.[23] Weather-related natural disasters such as storms and floods accounted for the vast majority of the 23.7M natural disaster (94%) driven internal displacements worldwide, underlining the threat of climate change.

NTD programmes need to better understand the burden and the extent to which these are singular or multiple intersecting challenges (Figure 1) to help to develop bespoke strategies and direct resources appropriately. This is important as some countries will experience more conflict- than climate, others more climate- than conflict-, or a complex combination of both conflict-climate.

Figure 1 here

Figure 1. Intersecting challenges of conflict, climate, and related internal displacement

We conducted an observational ecological study to better understand the burden of conflict-, violence- and natural disaster-related internal displacements in NTD endemic countries, and the extent to which these are singular or multiple intersecting challenges, to provide a basis from which bespoke strategies could be developed and resource allocation could be appropriately directed. We focused on countries in the World Health Organization (WHO) African region that are endemic for at least one of the five NTDs requiring preventive chemotherapy as a main intervention: lymphatic filariasis, onchocerciasis, schistosomiasis, soil-transmitted helminths, and trachoma.

For peer review only - http://bmjopen.bmj.com/site/about/guidelines.xhtml

METHODS

Study design

An observational ecological study design was used to examine the NTD, population and internal displacement factors and outcomes across different countries.

Neglected Tropical Disease and population data sources, classifications, and stratification

The NTD and population data were obtained from the Expanded Special Project for Elimination of NTDs (ESPEN) data portal, which collates data from national programmes and stakeholders on the various activities related to the implementation of MDA and/or morbidity management and disability (MMDP).[24] For each country, we summarised i) the number of NTDs requiring preventive chemotherapy and ii) the estimated country population based on 2020 data.

We then classified the countries endemic with four or five NTDs as '*High NTD number*' to reflect the complexity of managing many diseases and programmes, while the other countries were classified as '*Mod(erate)-Low NTD number*.' Second, we classified the ten countries with the highest populations (as an arbitrary threshold) as *'High population'* and others as '*Mod-Low population*'. Third, we combined the various classifications to develop four distinct categories, as shown here, and allocated them accordingly to each country.

NTD-population categories

- *High NTD number + High population*
- *High NTD number + Mod-Low population*
- *Mod-Low NTD number + High population*
- *Mod-Low NTD number + Mod-Low population*

Internal displacement data sources, classifications, and stratification

The data on internal displacements were obtained from the IDMC data portal, which monitors and collates country-specific data on internal displacements associated with conflict and generalised violence, and natural disasters (weather-related and geophysical) from a variety of sources.[23] The IDMC refers to internal displacement as the total number of movements that have taken place within a defined period e.g., between 1st January and 31st December 2021, and aims to provide a comprehensive cumulative figure, which may include people being displaced once or several times.

Conflict displacements are referred to as the total number of internal displacements reported as a result of conflict and/or violence events in a reporting year, and natural disaster displacements as the total number of internal displacements reported as a result of natural disasters in a reporting

For peer review only

year. For this paper focusing on the concept of climate, the related internal displacements are referred to as natural disasters internal displacements as defined by IDMC and only include countries with reported data for 2021.

For each country, we summarised the number of conflict-and natural disaster- internal displacements based on 2021 reported data[23] and calculated crude rates of internal displacement per 100,000 population based on ESPEN population data.[24] Countries with no conflict and climate reported data were not included in the analysis.

We then classified the 10 countries with the highest number and the highest rates of conflict-or disaster internal displacement as '*High conflict*' or '*High disaster*' and others as '*Mod-Low conflict*' or '*Mod-Low disaster*'. Second, the conflict and natural disaster classifications were then combined to develop four distinct categories each for numbers and rates as shown here and allocated to each country.

Displacement numbers and rate categories

- *High conflict + High disaster*
- *High conflict + Mod-Low disaster*
- *Mod-Low conflict + High disaster*
- *Mod-Low conflict + Mod-Low disaster*

Combined categories

All NTD, population and internal displacement categories were then combined to determine the overall patterns, and to help identify countries with high risk or vulnerability. For this paper, we highlighted selected high-risk combinations, which included the countries with a *High NTD number + High population* and a *High NTD number + Mod-Low population.*

Drivers of natural disaster internal displacement

To better understand the drivers of natural disaster internal displacement, we summarised the available IDMC data on hazard types in the countries with the highest natural disaster internal displacement numbers and rates in 2021. Hazard types were categorised as climate or weather-related (drought, flood, extreme temperature, storm, wet mass movement, and wildfire) and geophysical (earthquake, volcanic eruption, dry mass movement). The equivalent data for conflict internal displacement in 2021 were not available from this source and therefore not included in this paper.

For peer review only - http://bmjopen.bmj.com/site/about/guidelines.xhtml

Data management and mapping

The NTD, population and internal displacement data and related classifications and risk categories for each country were compiled into a Microsoft Excel file for descriptive analysis and mapping. Maps for all classifications and stratifications were created using mapping software ArcGIS 10.8.1 (ESRI, Redland CA) and national administrative boundaries from the ESPEN data portal.[24]. First, NTD, population, and international displacement individual classifications (e.g., *High, Mod-Low*) were mapped. Second, the different categories (e.g.., *High+High, High+Mod-Low, Low+Low*) of the different factors were mapped to highlight the variety of risk combinations. Finally, the countries with high-risk combinations for all factors (e.g., *High+High+High+High)* were mapped to highlight the countries with high risk or vulnerability.

Data limitations

We acknowledge that the use of open access data sources may have limitations if they are incomplete, and that our focus on a few selected data sources may cause unforeseen biases.

Patient and public involvement

No patient and/or members of the public were included in this study.

RESULTS

In total, 45 countries in the WHO African region had data on NTDs and populations (excluding Mauritius, Seychelles), and 32 countries reported data on conflict and/or natural disaster internal displacements; 16 countries reported both conflict and natural disaster; 15 countries reported natural disaster and one country reported conflict only internal displacement for 2021.

Neglected Tropical Diseases and population at risk

The total population in the 45 countries with data on NTDs was 1.1 billion. Thirty-one countries had a high number of NTDs: 12 countries endemic with five NTDs and 19 countries endemic with four NTDs (Figure 2A). The 10 countries with the highest populations are shown in Figure 2B, and of these, eight had both *High NTD number + High population* (Figure 2C) including (in descending order): Nigeria (est. 197.4M), Democratic Republic of Congo (DRC; 105.7M), Ethiopia (105.7M), Tanzania (mainland and Zanzibar - 55.9M), Kenya (52.0M), Uganda (41.0M), Angola (31.6M), Mozambique (29.9M) – totalling more than 619M people (Table 1).

Twelve countries had *Mod-Low NTD number + Mod-Low population* (Botswana, Cape Verde, Comoros, Eritrea, Eswatini, Lesotho, Mauritania, Namibia, Rwanda, Sao Tome and Principe, The

For peer review only

Gambia, Togo), while 24 countries had a combination of *High NTD number + Mod-Low population* and one country (South Africa) had a *Mod-Low NTD number + High population* (Figure 2C).

Figure 2 here

Figure 2. Summary maps of Neglected Tropical Diseases, populations, and internal displacement numbers, rates classifications and risk combinations

Conflict and natural disaster internal displacement numbers and rates

Numbers: The total number of internal displacements in 2021 in the 31 high NTD endemic countries was 2.2M. The conflict displacement classifications for each country are shown in Figure 2D; the *High conflict* displacement numbers ranged from 0.1M-5.1M across 10 countries: Ethiopia (5.14M), DRC (2.71M), Burkina Faso (0.68M), Central African Republic (CAR; 0.50M), South Sudan (0.43M), Mali (0.25M), Nigeria (0.38M), Mozambique (0.19M), Cameroon (0.13M) and Niger (0.11M); the *Mod-Low Conflict* numbers across seven countries ranged from 0.94M – 0.04M.

The natural disaster displacement classifications are shown in Figure 2E; the *High Disaster* numbers ranged from 0.24M-0.89M across 10 countries, reported in DRC (0.89M), South Sudan (0.51M), Ethiopia (0.24M), Niger (0.12M), Burundi (0.09M), Tanzania (0.05M), Uganda (0.05M), Mozambique (0.04M), Kenya (0.04M), Nigeria (0.02M); the *Mod-Low disaster* numbers in 20 countries ranged from 255 – 0.02M.

Six countries had both *High conflict + High disaster* internal displacement numbers, including (in descending order) Ethiopia (5.38M combined), DRC (3.60M), South Sudan (0.93M), Nigeria (0.40M), Mozambique (0.23M) and Niger (0.23M) (Figure 2F) – totalling more than 10.8M conflict and natural disaster combined internal displacements in 2021 (Table 1).

Table 1 here

Rates: The conflict internal displacement classifications are shown in Figure 2G; the *High conflict* displacement rates (Table 1) ranged from 252.7 – 9020.3 per 100,000 population, reported in CAR (9020.3 per 100,000), Ethiopia (4867.1), South Sudan (3255.6), Burkina Faso (3176.4), DRC (2564.7), Mali (1187.2), Mozambique (625.3), Cameroon (504.0), Niger (475.1), Chad (252.7); the *Mod-Low conflict* displacement rates ranged from 0.082 - 190.2 per 100,000.

For peer review only - http://bmjopen.bmj.com/site/about/guidelines.xhtml

The natural disaster internal displacement classifications are shown in Figure 2H; the *High disaster* displacement rates (Table 1) ranged from 144.1-3832.6 per 100,000 population, reported in South Sudan (3832.6 per 100,000), DRC (839.8), Burundi (754.7), Niger (507.6), CAR (429.3), Sao Tome and Principe (237.8), Ethiopia (227.2), Namibia (165.0), Mozambique (145.5), Chad (144.1); the *Mod-Low disaster* displacement rates ranged from 3.2 - 121.4 per 100,000.

Seven countries had both *High conflict + High disaster* internal displacement rates, including (in descending order) CAR (9449.7 combined), South Sudan (7088.1), Ethiopia (5094.3), DRC (3404.5), Niger (982.7), Mozambique (770.8) and Chad (398.8) (Figure 2I).

For peer review only

Table 1. **Summary of populations, and internal displacement data and high-risk combinations in selected countries** with high number of NTDs

Location‡	Population *	Internal displacement numbers†			Internal displacement rates per 100,000 population			Combined classifications
**Country**	**Population**	**Conflict No.**	**Natural Disaster No.**	**Conflict Disaster No.**	**Conflict rate**	**Natural Disaster rate**	**Conflict Disaster rate**	**NTD, Population Conflict, Natural Disaster**
**High NTDs + High population**								
DRC	105.7M	2,711,783	888,000	3.60M	2,564.7	839.8	3,404.5	High NTD-population+ conflict-disaster
Ethiopia	105.7M	5,142,356	240,009	5.38M	4867.1	227.2	5,094.3	High NTD-population+ conflict-disaster
Mozambique	29.9M	187,057	43,512	0.23M	625.3	145.5	770.8	High NTD-population+ conflict-disaster
Nigeria	197.4M	375,552	24,366	0.40M	190.2	12.3	202.6	
Tanzania	55.9M	0	46,707	0.05M	0	85.6	85.6	
Kenya	52.0M	4,634	36,442	0.04M	8.9	70.1	79.0	
Uganda	41.0M	1,278	46,690	0.05M	3.1	113.8	116.9	
Angola	31.6M	0	21727	0.02M	0	68.8	68.8	
**Summary**	**619M+**	**8,422,660**	**1,347,453**	**9.77M**	**0-4,867.1**	**12.3-839.8**	**68.8 – 5,094.3**	
**High NTD number + Mod-Low population**								
South Sudan	13.2M	429,432	505,544	0.93M	3255.6	3,832.6	7,088.1	High NTD-conflict-disaster
Niger	23.2M	110,203	117,747	0.23M	475.1	507.6	982.7	High NTD-conflict-disaster
Burkina Faso	21.5M	682,245	0	0.68M	3176.4	0	3176.4	High NTD - conflict
Cameroon	26.1M	131,452	1,764	0.13M	504.0	6.8	510.8	High NTD– conflict
CAR	5.5M	495,600	23,589	0.52M	9,020.3	429.3	9,449.7	High NTD - conflict
Mali	21.0M	249,174	5,994	0.26M	1,187.2	28.6	1,215.7	High NTD - conflict
Burundi	11.5M	94	86,885	0.09M	0.8	754.7	755.5	High NTD - disaster
Chad	16.8M	42,355	24,144	0.07M	252.7	144.1	398.8	
**Summary**	**138.8M**	**2,140,555**	**765,667**	**2.91M**	**0.8-9,020.3**	**0 – 3,832.6**	**398.8 – 9,449.7**	

‡ All countries have a high number of NTDs
*ESPEN data 2021[24]
† IDMC data 2019-2021[23]
Shading represents countries with a classified High NTD number, High population, or *High Conflict* and/or *High Natural disaster* internal displacement numbers and/or rates

For peer review only - http://bmjopen.bmj.com/site/about/guidelines.xhtml

Risk combinations

Of the countries with *High NTD number + High population*, we found that DRC, Ethiopia, Mozambique, and Nigeria had *High conflict + High disaster* displacement numbers (Figure 2J), and DRC, Ethiopia, and Mozambique had *High conflict + High disaster* displacement rates (Figure 2K) in 2021. Collectively, these three countries reported more than 241M people at risk of four to five NTDs and 9.2M conflict and/or natural disaster internal displacements in 2021 alone; these may be considered the most at risk or vulnerable countries in the African region. Nigeria was found to have *High conflict + High disaster* displacement numbers, while Tanzania, Kenya, and Uganda had among the highest *High disaster* displacement numbers (Table 1).

Of the countries with high NTD numbers and displacement, we found South Sudan and Niger to have both *High conflict + High disaster* number and rates, while Burkina Faso, Cameroon, CAR, and Mali had both *High conflict* numbers and rates, and Burundi had both *High disaster* displacement numbers and rates in 2021 (Table 1).

Drivers of natural disaster internal displacement

The number of reported natural disaster hazard events in 2021 varied across countries, with the highest frequency reported in Burundi (n=80), Uganda (n=60) and South Sudan (n=31), and the highest number of hazard-related internal displacements reported in DRC (0.89M), South Sudan (0.51M), and Ethiopia (0.24M) (Table 2). In all countries, weather-related hazards were most common with floods the dominant hazard type, accounting for around half (55.2%) of all natural disaster displacements across 12 countries, except DRC where a volcanic disruption led to 0.6M displacements, and in Mozambique where a storm led to 0.04M displacements. In total, 150 flood events across 11 countries caused 0.85M natural disaster hazard related internal displacements. Other causes of internal displacement included mass wet or dry movement and wildfires but affected far fewer numbers.

For peer review only

Table 2. Summary of natural disaster internal displacements by hazard type in selected countries

Country	Total Populat ion.	Total number (No.) of Natural Disaster Displacements	Total rate of Natural Disaster Displacements	Total No. hazard events	No. of natural disaster displacements by hazard type (No. of hazard events)			
					**Flood**	**Drought**	**Storm**	**Other***
**High NTD number, High population, High climate numbers or rates**								
DRC	105.7M	0.89M	2564.7	21	0.26M (n=10)		0.002M (n=8)	0.6M (n=3)
Ethiopia	105.7M	0.24M	839.8	7	0.19M (n=4)	0.05M (n=1)		0.00001 (n=2)
Mozambique	29.9M	0.04M	145.5	2			0.04M (n=2)	
Nigeria	197.4M	0.02M	12.3	31	0.02M (n=31)			
Tanzania	55.0M	0.05M	85.6	6	0.02M (n=2)		0.03M (n=4)	
Kenya	52.0M	0.04M	70.1	3	0.04M (n=2)		0.000M (n-1)	
Uganda	41.0M	0.05M	113.8	60	0.04M (n=24)		0.005M (n=28)	0.001M (n=8)
**Summary**	**586.7M**	**1.33**	**12.3 – 2564.7**	**130**	**0.57M (n=73)**	**0.05M (n=1)**	**0.08 (n=43)**	**0.60 (n=13)**
**High NTD number, Mod-Low population, high natural disaster numbers or rates**								
South Sudan	13.2M	0.05M	3832.6	39	0.05M (n=39)			
Niger	23.2M	0.12M	507.6	1	0.12M (n=1)			
CAR	5.5M	0.02M	429.3	16	0.02 (n=16)			
Burundi	11.5M	0.26M	754.7	80	0.07M (n=15)		0.013M (n=63)	0.0002 (n=1)
Chad	16.8M	0.02M	144.1	6	0.02 (n=6)			
**Summary**	**70.2M**	**0.47M**	**144.1 – 3832.6**	**142**	**0.28 (n=77)**		**0.013M (n=63)**	**0.0002 (n=1)**

Data source iDMC (numbers rounded)[23]
*Other category included – wet or dry mass movement and/or wildfires

For peer review only

DISCUSSION

This paper highlights that many African countries have high numbers of NTDs, which require a great deal of coordination and logistics in-country to implement routine NTD programming.[24] In the eight most populous endemic countries, we quantified that more than half a billion people will need multiple NTD interventions and long-term surveillance activities in the next decade. Routine NTD programming has been and will be negatively impacted by large population displacements, which adds to the existing difficulties of determining accurate population numbers in countries where there is a lack of regular census data.[25,26] The ESPEN data portal provides population estimates[24] and could start to collate data on internal displacements from various sources, including NTD programme managers who are experiencing the challenges first hand and may provide more detailed information and invaluable insights into these problems.

Alarmingly, here we show in just one year that Ethiopia, DRC, South Sudan, Nigeria, Mozambique, and Niger reported more than 10M internal conflict and natural disaster internal displacements and have among them the highest conflict and natural disaster rates per population. If current trends continue or increase, these countries are at high risk of not reaching the NTD road map targets and will require significant technical and financial support from stakeholders if global goals are to be met.[1] This is critical as these six countries have among the lowest levels of human development in the world,[27] and may not be able to access and mobilise in-country resources needed to accelerate programmatic action, intensify cross-cutting approaches, and change operating models to facilitate enhanced country ownership as set out in the NTD road map 2021-2030.[1]

We limited our analysis to one internal displacement data source but advocate that future research should aim to triangulate information from other data sources to build a comprehensive picture of the situation. A further limitation was that we applied simple classifications and risk combinations to the NTD, population, and internal displacement figures to allow us to initially focus on countries most affected as a means of understanding the magnitude of these complex challenges. This does not imply that countries with lower numbers or rates of NTDs, population, conflict and natural disaster internal displacements are less of a priority for NTD programmes, but rather the magnitude is different. All endemic countries in Africa and elsewhere will need support to address these challenges and future work should consider more advanced analysis applied at a subnational level and include additional compounding factors such as health infrastructure, community accessibility, and strength of the NTD programme.[15,20]

For peer review only - http://bmjopen.bmj.com/site/about/guidelines.xhtml

Further, future work also needs to consider the spatial-temporal patterns and geographical overlap of conflict and natural disaster driven displacement to identify hotspots, and determine the long-term implications and strategies needed for NTD programme activities. For example, if there is protracted conflict in an area, then programmes can adapt their methods of programmatic delivery, target non-conflict areas, or identify methods of treating displaced populations that have sought refuge in secure areas.[17] If flooding is increasingly predictable in an area, then programmes can adapt the timing of programme activities in that area or identify alternate treatment sites for those displaced by the flooding.

The African continent is predicted to continue experiencing negative effects from climate change due to increasing temperatures, rising sea-levels, cumulative failed rainy seasons, drought, and shrinking surface water.[8] These events and resulting food and water scarcity can feed into political instability, and exacerbate ongoing tensions thereby creating a vulnerability multiplier for those at risk of conflict, natural disasters, and NTDs. Climate change is an escalating security threat,[28–31] and an understanding of the key drivers is critical in Africa and elsewhere. Here we highlight that most natural disaster hazards were climate or weather-related events, especially flooding, which led to nearly one million displacements in 11 African countries in one year alone. However, the impact of these events on disease transmission dynamics in highly endemic areas, and how they affect programmes, is largely unknown as very little research has been conducted in Africa and elsewhere.[4–6]

Moving forward NTD programmes will need to engage a range of other stakeholders that are working towards addressing and/or mitigating the impact of conflict and/or climate change at an international and national level. [32] This will help to identify country specific needs and create opportunities for cross-sector partnerships, good coordination, and the potential co-production and co-implementation of effective strategies.

Recommendations

We advocate for a “call to action” to encourage policy makers, programme managers, researchers, implementing partners and donors to develop, implement, and evaluate a set of strategies to better assess NTD burden in areas at risk or experiencing conflict and climate related natural disasters, and deliver interventions that are urgently required. Here we focus on African countries, but the same methodologies may be applied to different WHO regions to understand the distinct and common challenges. There is a need to build on core principles and adapted survey strategies already being

For peer review only

developed and implemented by experienced teams that may address these special populations and problems.[14–16,33,34] It will also be important to learn from countries that have implemented efficient and timely responses during conflicts and disasters to internally displaced populations through cross-sectoral collaboration.

Though national capacity to respond to humanitarian emergencies can be limited, as a first step we suggest an overarching set of recommendations that include the development of preferred practices; data platform; decision support tool, advocacy and communication and implementation research strategies as outlined in Box 1.

Box 1. Overarching recommendations to address complex challenges in NTD endemic countries

- PREFERRED PRACTICES: A set of informed preferred practice documents outlining potential step-by step processes and methods to address singular or multiple intersecting conflict- climate-displacement challenges that impede NTD programmatic activities.
- DATA PLATFORM: A central data portal that combines data for each country on: NTD burden, population size, internal and cross-border displacement numbers (by conflict, natural disaster, and hazard type categorisation) and administrative boundaries.
- DECISION SUPPORT TOOL: A practical online tool that facilitates the national NTD programme teams to input and analysis conflict-climate-displacement data to assess risk and support programmatic decision-making at implementation unit or district level.
- ADVOCACY AND COMMUNICATION. An advocacy and communication plan with goals to document and inform national and international stakeholders of the key threats and success stories related to the conflict-climate-displacement challenges that NTD programmes confront.
- IMPLEMENTATION RESEARCH: A strategic research agenda outlining the range of conflict- climate-displacement challenges, and the potential research questions, methodological approaches, and funding opportunities that could be drawn on by NTD programmes and academic partners.

CONCLUSION

This paper highlights the urgent need for health policies and guidelines to address the escalating risks of conflict, climate change-induced natural disasters, and associated internal displacement of populations in NTD endemic countries in Africa. We have shown that publicly available data sources

For peer review only - http://bmjopen.bmj.com/site/about/guidelines.xhtml

could potentially be used by national policy makers and NTD programmes as a first step to better understanding the intersecting challenges and their level of risk and vulnerability. This would allow them to develop risk assessments and mitigation strategies that may be tailored to the local context and health needs of internally displaced persons, given that the types and burden of NTDs, and the causes of internal displacement, vary within and between countries. There is a need to build new stakeholder collaborations and a collective agenda for these neglected conflict-climate-displacement challenges, so that NTD programmes can meet the 2021-2030 road map targets and 2030 Agenda for Sustainable Development and ensure that no one is left behind.

CONTRIBUTORS

LAK-H contributed to the conception, methodology, formal analysis, writing-original draft. EH-E, and AMS contributed to the interpretation, writing of the recommendations, writing review and editing. JW and FA contributed to the interpretation, writing review and editing. All authors approved the final version. All authors are members of the Neglected Tropical Diseases Non-Governmental Organisation Network (NNN), Conflict and Humanitarian Emergency (C&HE) Cross-Cutting Group - Mapping Task Team https://www.ntd-ngonetwork.org/cross-cutting-groups/conflict-and-humanitarian-emergencies.

DATA AVAILABILITY STATEMENT

All data relevant to this study are included in the article or available in the public, open access repositories.

ETHICS STATEMENT

Patient consent for publication

This study does not involve human participants and ethical approval was not required.

COMPETING INTERESTS

None declared

FUNDING

The work was not funded by a specific grant. The University of Liverpool provided funding for the open access publishing cost.

For peer review only

REFERENCES

1 World Health Organization. Ending the neglect to attain the sustainable development goals: a road map for neglected tropical diseases 2021–2030. Geneva: World Health Organization, 2020 https://apps.who.int/iris/handle/10665/338565 (accessed Aug 4, 2022).

2 Bangert M, Molyneux DH, Lindsay SW, Fitzpatrick C, Engels D. The cross-cutting contribution of the end of neglected tropical diseases to the sustainable development goals. *Infectious Diseases of Poverty* 2017; **6**: 73.

3 United Nations.Department of Economic and Social Affairs. Sustainable Development Goals. Sustainable Development. https://sdgs.un.org/goals (accessed Dec 14, 2022).

4 Tidman R, Abela-Ridder B, de Castañeda RR. The impact of climate change on neglected tropical diseases: a systematic review. *Transactions of The Royal Society of Tropical Medicine and Hygiene* 2021; **115**: 147–68.

5 Bryson JM, Bishop-Williams KE, Berrang-Ford L, *et al.* Neglected Tropical Diseases in the Context of Climate Change in East Africa: A Systematic Scoping Review. *The American Journal of Tropical Medicine and Hygiene* 2020; **102**: 1443–54.

6 Booth M. Chapter Three - Climate Change and the Neglected Tropical Diseases. In: Rollinson D, Stothard JR, eds. Advances in Parasitology. Academic Press, 2018: 39–126.

7 Eckstein D, Künzel V, Schäfer L. Global Climate Risk Index 2021 Who Suffers Most Extreme Weather Events? Weather-Related Loss Events in 2019 and 2000-2019. 2021.

8 World Meteorological Organization. State of Climate Africa 2021. 2022 https://library.wmo.int/index.php?lvl=notice_display&id=22125#.Y5l2B-zP2lM (accessed Dec 14, 2022).

9 Zielinski C. COP27 climate change conference: urgent action needed for Africa and the world. *BMJ Global Health* 2022; **7**: e010965.

10 Palik J, Rustad SA, Methi F. Conflict Trends in Africa, 1989–2019. .

11 Raleigh C, Linke A, Hegre H, Karlsen J. Introducing ACLED: An Armed Conflict Location and Event Dataset: Special Data Feature. *Journal of Peace Research* 2010; **47**: 651–60.

12 Yaya G. Lessons learned in the implementation of programmes to eliminate trachoma within conflict zones. *Trans R Soc Trop Med Hyg* 2022; : trac061.

13 Du RY, Stanaway JD, Hotez PJ. Could violent conflict derail the London Declaration on NTDs? *PLoS Negl Trop Dis* 2018; **12**: e0006136.

14 McPherson, Scott. Working outside our comfort zone: Fighting NTDs in conflict areas. Working outside our comfort zone: Fighting NTDs in conflict areas. https://iamtropmed.org/blog/2019/11/21/working-outside-our-comfort-zone-fighting-ntds-in-conflict-areas (accessed March 19, 2023).

15 Harvey D, Shu'aibu J, Debam MT, Aba AK, Torres-Vitolas CA. How can the neglected tropical disease community be inclusive and equitable in programme delivery? Reaching refugees and internally displaced persons through integrating a 'leave no one behind' approach. *Int Health* 2022; **14**: ii33–7.

For peer review only

16 Neglected Tropical Disease NGO Network (NNN). Conflict and Humanitarian Emergencies. https://www.ntd-ngonetwork.org/cross-cutting-groups/conflict-and-humanitarian-emergencies (accessed Aug 24, 2022).

17 Kelly-Hope LA, Sanders AM, Harding-Esch E, *et al.* Complex emergencies and the control and elimination of neglected tropical diseases in Africa: developing a practical approach for implementing safe and effective mapping and intervention strategies. *Confl Health* 2021; **15**: 18.

18 Kelly-Hope LA, Molyneux DH. Quantifying conflict zones as a challenge to certification of Guinea worm eradication in Africa: a new analytical approach. *BMJ Open* 2021; **11**: e049732.

19 Cantor D, Swartz J, Roberts B, *et al.* Understanding the health needs of internally displaced persons: A scoping review. *J Migr Health* 2021; **4**: 100071.

20 Adams MW, Sutherland EG, Eckert EL, Saalim K, Reithinger R. Leaving no one behind: targeting mobile and migrant populations with health interventions for disease elimination—a descriptive systematic review. *BMC Med* 2022; **20**: 172.

21 World Health Organization. Summary of global update on implementation of preventive chemotherapy against NTDs in 2020. 2021.

22 Bockarie MJ, Kelly-Hope LA, Rebollo M, Molyneux DH. Preventive chemotherapy as a strategy for elimination of neglected tropical parasitic diseases: endgame challenges. *Philos Trans R Soc Lond B Biol Sci* 2013; **368**: 20120144.

23 Internal Displacement Monitoring Centre (IDMC). Global Internal Displacement Database. Data (2022). https://www.internal-displacement.org/database/displacement-data.

24 World Health Organization. Expanded Special Project for Elimination of Neglected Tropical Diseases (ESPEN). 2022. https://espen.afro.who.int/ (accessed Aug 9, 2022).

25 Carlin, Alexandra. Why we should reconsider population data for disease control and public health programmes. BMC BugBitten. 2021; published online Nov 26. https://blogs.biomedcentral.com/bugbitten/2021/11/26/population-data-disease-control-public-health/ (accessed Oct 10, 2022).

26 Ratnayake R, Abdelmagid N, Dooley C. What we do know (and could know) about estimating population sizes of internally displaced people. *Journal of Migration and Health* 2022; **6**: 100120.

27 United Nations Develop Programme (UNDP). Human Development Report 2021-22: Uncertain Times, Unsettled Lives: Shaping our Future in a Transforming World. New York, 2022 https://hdr.undp.org/data-center/human-development-index#/indicies/HDI (accessed Oct 10, 2022).

28 United Nations Environment Programme (UNEP). Climate change and security risks. Disasters and conflicts. 2022. https://www.unep.org/explore-topics/disasters-conflicts/what-we-do/disaster-risk-reduction/climate-change-and-security (accessed Oct 13, 2022).

29 Brown O, Hammill A, Mcleman R. Climate change as the ‘new’ security threat: implications for Africa. *International Affairs* 2007; **83**: 1141–54.

30 Evans MS, Munslow B. Climate change, health, and conflict in Africa’s arc of instability. *Perspect Public Health* 2021; **141**: 338–41.

For peer review only - http://bmjopen.bmj.com/site/about/guidelines.xhtml

31 Mach KJ, Kraan CM, Adger WN, *et al.* Climate as a risk factor for armed conflict. *Nature* 2019; **571**: 193–7.

32 United Nations Office for the Coordination of Humanitarian Affairs (OCHA). Humanitarian System/Clusters/What is the Cluster approach? https://www.humanitarianresponse.info/en/coordination/clusters/what-cluster-approach (accessed March 19, 2023).

33 Sanders AM, Abdalla Z, Elshafie BE, *et al.* Prevalence of trachoma within refugee camps serving South Sudanese refugees in White Nile State, Sudan: Results from population-based surveys. *PLoS Negl Trop Dis* 2019; **13**: e0007491.

34 The International, Coalition for Trachoma Control, (ICTC). THE END IN SIGHT: From collaboration to elimination, strategic plan 2022–2030. 2022; published online May. https://www.trachomacoalition.org/sites/default/files/content/resources/files/ICTC%20Strategic%20Plan.pdf (accessed Oct 10, 2022).

For peer review only

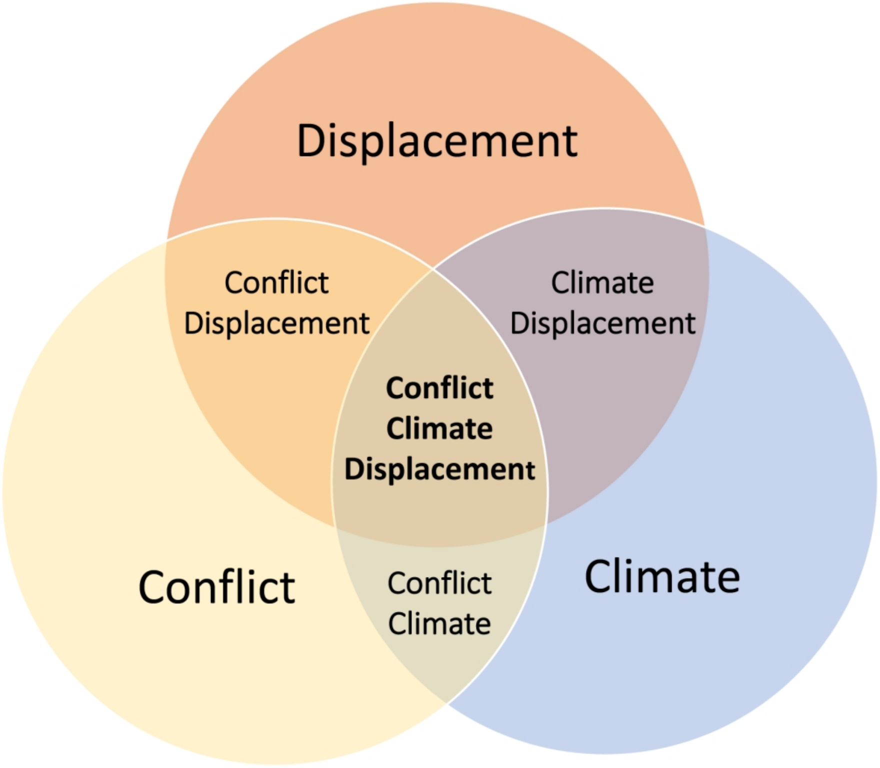

164x147mm (300 x 300 DPI)

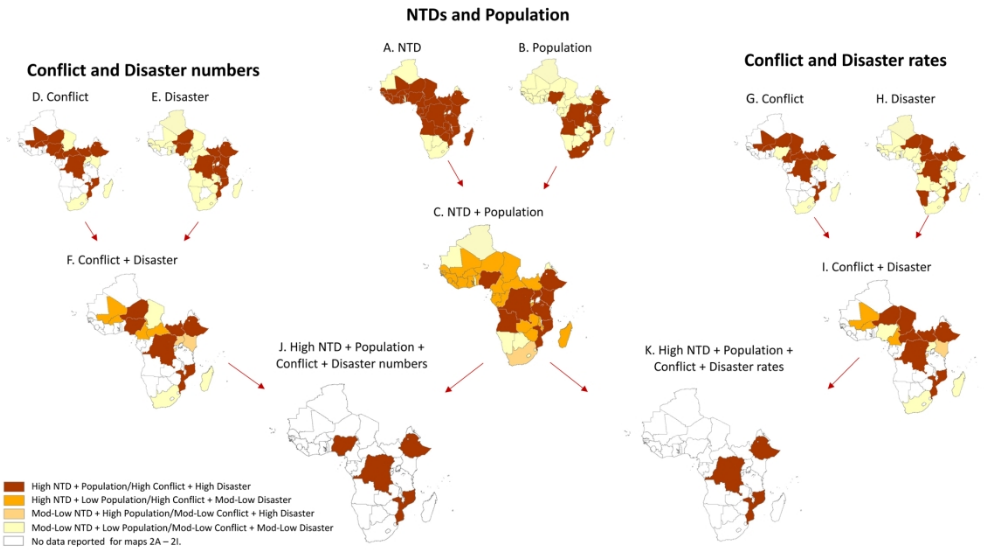

170x95mm (300 x 300 DPI)

STROBE Statement—Checklist of items that should be included in reports of ***cross-sectional studies***

	Item No	Recommendation	Page No
**Title and abstract**	1	(*a*) Indicate the study's design with a commonly used term in the title or the abstract	2
		(*b*) Provide in the abstract an informative and balanced summary of what was done and what was found	2
**2Introduction**			
Background/rationale	2	Explain the scientific background and rationale for the investigation being reported	4-5
Objectives	3	State specific objectives, including any prespecified hypotheses	5
**Methods**			
Study design	4	Present key elements of study design early in the paper	5
Setting	5	Describe the setting, locations, and relevant dates, including periods of recruitment, exposure, follow-up, and data collection	6
Participants	6	(*a*) Give the eligibility criteria, and the sources and methods of selection of participants	6-7
Variables	7	Clearly define all outcomes, exposures, predictors, potential confounders, and effect modifiers. Give diagnostic criteria, if applicable	6-7
Data sources/ measurement	8*	For each variable of interest, give sources of data and details of methods of assessment (measurement). Describe comparability of assessment methods if there is more than one group	*6-7*
Bias	9	Describe any efforts to address potential sources of bias	6
Study size	10	Explain how the study size was arrived at	6-7
Quantitative variables	11	Explain how quantitative variables were handled in the analyses. If applicable, describe which groupings were chosen and why	6-7
Statistical methods	12	(*a*) Describe all statistical methods, including those used to control for confounding	N/A
		(*b*) Describe any methods used to examine subgroups and interactions	6-7
		(*c*) Explain how missing data were addressed	6
		(*d*) If applicable, describe analytical methods taking account of sampling strategy	6-7
		(*e*) Describe any sensitivity analyses	N/A
**Results**			
Participants	13*	(a) Report numbers of individuals at each stage of study—eg numbers potentially eligible, examined for eligibility, confirmed eligible, included in the study, completing follow-up, and analysed	N/A
		(b) Give reasons for non-participation at each stage	N/A
		(c) Consider use of a flow diagram	Figure 2
Descriptive data	14*	(a) Give characteristics of study participants (eg demographic, clinical, social) and information on exposures and potential confounders	8-12
		(b) Indicate number of participants with missing data for each variable of interest	N/A
Outcome data	15*	Report numbers of outcome events or summary measures	8-12

For peer review only

Main results	16	(*a*) Give unadjusted estimates and, if applicable, confounder-adjusted estimates and their precision (eg, 95% confidence interval). Make clear which confounders were adjusted for and why they were included	N/A
		(*b*) Report category boundaries when continuous variables were categorized	8-12
		(*c*) If relevant, consider translating estimates of relative risk into absolute risk for a meaningful time period	N/A
Other analyses	17	Report other analyses done—eg analyses of subgroups and interactions, and sensitivity analyses	8-12
**Discussion**			
Key results	18	Summarise key results with reference to study objectives	13
Limitations	19	Discuss limitations of the study, taking into account sources of potential bias or imprecision. Discuss both direction and magnitude of any potential bias	13-14
Interpretation	20	Give a cautious overall interpretation of results considering objectives, limitations, multiplicity of analyses, results from similar studies, and other relevant evidence	13-14
Generalisability	21	Discuss the generalisability (external validity) of the study results	13-14
**Other information**			
Funding	22	Give the source of funding and the role of the funders for the present study and, if applicable, for the original study on which the present article is based	16

*Give information separately for exposed and unexposed groups.

Note: An Explanation and Elaboration article discusses each checklist item and gives methodological background and published examples of transparent reporting. The STROBE checklist is best used in conjunction with this article (freely available on the Web sites of PLoS Medicine at http://www.plosmedicine.org/, Annals of Internal Medicine at http://www.annals.org/, and Epidemiology at http://www.epidem.com/). Information on the STROBE Initiative is available at www.strobe-statement.org.

For peer review only

For peer review only - http://bmjopen.bmj.com/site/about/guidelines.xhtml

BMJ Open

Conflict-climate-displacement: a cross-sectional ecologic study determining the burden, risk and need for strategies for Neglected Tropical Disease programmes in Africa.

Journal:	*BMJ Open*
Manuscript ID	bmjopen-2023-071557.R2
Article Type:	Original research
Date Submitted by the Author:	02-May-2023
Complete List of Authors:	Kelly-Hope, Louise; University of Liverpool<br>Harding-Esch, Emma; London School of Hygiene & Tropical Medicine, Clinical Research Department<br>Willems, Johan; CBM Christoffel-Blindenmission Christian Blind Mission e.V.<br>Ahmed, Fatima; The MENTOR Initiative<br>Sanders, Angelia; The Carter Center
**Primary Subject Heading**:	Global health
Secondary Subject Heading:	Epidemiology, Health policy, Infectious diseases, Public health
Keywords:	Public health < INFECTIOUS DISEASES, Epidemiology < TROPICAL MEDICINE, Epidemiology < INFECTIOUS DISEASES, Neglected Diseases

SCHOLARONE™
Manuscripts

I, the Submitting Author has the right to grant and does grant on behalf of all authors of the Work (as defined in the below author licence), an exclusive licence and/or a non-exclusive licence for contributions from authors who are: i) UK Crown employees; ii) where BMJ has agreed a CC-BY licence shall apply, and/or iii) in accordance with the terms applicable for US Federal Government officers or employees acting as part of their official duties; on a worldwide, perpetual, irrevocable, royalty-free basis to BMJ Publishing Group Ltd ("BMJ") its licensees and where the relevant Journal is co-owned by BMJ to the co-owners of the Journal, to publish the Work in this journal and any other BMJ products and to exploit all rights, as set out in our licence.

The Submitting Author accepts and understands that any supply made under these terms is made by BMJ to the Submitting Author unless you are acting as an employee on behalf of your employer or a postgraduate student of an affiliated institution which is paying any applicable article publishing charge ("APC") for Open Access articles. Where the Submitting Author wishes to make the Work available on an Open Access basis (and intends to pay the relevant APC), the terms of reuse of such Open Access shall be governed by a Creative Commons licence – details of these licences and which Creative Commons licence will apply to this Work are set out in our licence referred to above.

Other than as permitted in any relevant BMJ Author's Self Archiving Policies, I confirm this Work has not been accepted for publication elsewhere, is not being considered for publication elsewhere and does not duplicate material already published. I confirm all authors consent to publication of this Work and authorise the granting of this licence.

For peer review only

Journal Article

TITLE: Conflict-climate-displacement: a cross-sectional ecologic study determining the burden, risk and need for strategies for Neglected Tropical Disease programmes in Africa.

AUTHORS: Louise A. Kelly-Hope[1*], Emma Harding-Esch[2], Johan Willems[3], Fatima Ahmed[4], Angelia M. Sanders[5]

* Corresponding author: lkhope@liverpool.ac.uk

AFFILIATIONS: *Note; Authors are members of the Neglected Tropical Diseases Non-Governmental Organisation Network (NNN), Conflict and Humanitarian Emergency (C&HE) Cross-Cutting Group - Mapping Task Team.*

1. Institute of Infection, Veterinary & Ecological Sciences, University of Liverpool, UK
2. London School of Hygiene & Tropical Medicine, London, UK.
3. CBM, Christoffel-Blindenmission Christian Blind Mission e.V., Bensheim, Germany.
4. The MENTOR Initiative, Haywards Heath, UK
5. The Carter Center, Atlanta, USA.

For peer review only

ABSTRACT

Objectives: Complex challenges such as political instability, climate change, and population displacement are increasing threats to national disease control, elimination, and eradication programmes. The objective of this study was to determine the burden and risk of conflict and climate related internal displacements and the need for strategies for countries endemic with Neglected Tropical Diseases (NTDs).

Design, setting and outcome measures: A cross-sectional ecologic study was conducted including countries that are endemic with at least one of five NTDs requiring preventive chemotherapy in the African region. For each country, the number of NTDs, population size, and the number and rate per 100 000 of conflict-and natural disaster- internal displacements reported in 2021 were classified into high and low categories and used in unison to stratify and map the burden and risk.

Results: This analysis identified 45 NTD-endemic countries; eight countries were co-endemic with four or five diseases and had populations classified as "high" totalling more than 619 million people. We found 32 endemic countries had data on internal displacements related to conflict and disasters (n=16), disasters only (n=15) or conflict only (n=1). Six countries had both high conflict and disaster internal displacement numbers totalling more than 10.8 million people, and five countries had combined high conflict and disaster internal displacement rates, ranging from 770.8 to 7088.1 per 100 000 population. Weather-related hazards were the main cause of natural disaster displacements, predominately floods.

Conclusions: This paper presents a risk stratified approach to better understand the potential impact of these complex intersecting challenges. We advocate for a "call to action" to encourage national and international stakeholders to further develop, implement, and evaluate strategies to better assess NTD endemicity, and deliver interventions, in areas at risk of, or experiencing, conflict and climate disasters, in order to help meet the national targets.

For peer review only - http://bmjopen.bmj.com/site/about/guidelines.xhtml

STRENGTHS AND LIMITATIONS OF THIS STUDY

This study is the first to document the number of conflict- and natural disaster- related internal displacements in high Neglected Tropical Disease (NTDs) endemic countries in Africa.

The methodology employed is based on publicly available resources downloaded from the Expanded Special Project for Elimination of NTDs (ESPEN) data portal and the Internal Displacement Monitoring Centre (IDMC).

A new practical risk stratification approach is presented to help determine the intersecting challenges of NTDs and conflict- and natural disaster- related internal displacements, and highlights each country's unique combination of risk or vulnerability.

This information can be used for advocacy purposes to develop and implement mitigation strategies to help direct resources and enable effective NTD control and elimination programmes.

Future work needs to consider a range of data sources and spatial-temporal patterns to help identify hotspots, and determine the long-term implications and strategies needed for NTD programme activities.

For peer review only

INTRODUCTION

The Neglected Tropical Disease (NTD) road map 2021-2030 sets out aspiring global targets to prevent, control, eliminate or eradicate 20 NTDs in more than 100 endemic countries.[1] The roadmap highlights how reaching these goals can contribute to attaining the Sustainable Development Goals (SDGs),[2,3] and emphasises the need to accelerate programmatic action, intensify cross-cutting approaches, and change operating models and culture to facilitate country ownership. Importantly, it raises the need to address complex challenges such as climate change, political instability, and population movement and displacement, which threatens achieving the road map targets, and are increasing and intrinsically interlinked but have received little attention to-date.

Climate change can alter local ecology and disease dynamics.[4–6] It is expected to increase the intensity and frequency of extreme weather events and induce natural disasters such as floods and drought, which can restrict access to health care, limit water and food systems, cause outbreaks of climate-sensitive diseases, and lead to forced migration and internal displacement as living areas become inhabitable and livelihoods are destroyed.[7,8] The African continent is extremely vulnerable to climate change.[9] However there is a major knowledge gap associated with its impact on NTD transmission and programme activities across highly endemic countries.

Political instability can disrupt governance and escalate internal conflict and violence, and therefore restrict safe access to health care and potentially cause forced migration and internal displacement as people seek refuge. In Africa, the number of conflict events has increased dramatically in recent years[10,11] and impeded the effectiveness of many NTD programmes.[12–15] In response, practical approaches have been developed to address key issues such as safe and effective disease mapping and intervention strategies,[16–18] however more support to implement these at scale, and better understand the intersect with climate and complex humanitarian emergencies leading to displacement and how it affects vulnerable populations is urgently needed.[19]

External and internal displacement are driven by multiple causes and pose multiple risks for national programmes.[20] They can result in the introduction or recrudescence of infectious diseases, and the shortage of essential services such as health care, water and sanitation in areas with large unplanned population fluxes. These factors make it difficult for programmes to implement and assess the impact of interventions such as mass drug administration (MDA), which is being widely distributed as preventive chemotherapy for a number of NTDs.[1,21] The lack of specific protocols for distributing MDA to internally displaced populations and little understanding of the inhibiting factors that programmes face exacerbates the problem.

For peer review only - http://bmjopen.bmj.com/site/about/guidelines.xhtml

For peer review only

If the NTD road map 2021-2030 global targets are to be achieved, these challenges that NTD programmes face need to be at the forefront of national plans and stakeholder funding in the next decade. Currently, there are no formal health policies or guidelines on how national programmes may begin to document the burden, assess the impact, and develop strategies to overcome these pervasive threats, nor (to the best of our knowledge), specific funding streams exist for operational research and policy development. This is of significant concern and needs prioritising given that these challenges will drastically hinder progress, especially in Africa where many large, highly NTD endemic countries have additional programme challenges and are lagging behind key programmatic activities.[1,22]

The frequency of these displacement-related events in many NTD endemic countries is escalating, highlighting the need for urgent attention. The Internal Displacement Monitoring Centre (IDMC) highlights that conflict, violence and natural disasters caused approximately 38 million (M) internal displacements worldwide in 2021 with 80% of conflict and violence triggered displacements in sub-Saharan Africa alone.[23] Weather-related natural disasters such as storms and floods accounted for the vast majority of the 23.7M natural disaster (94%) driven internal displacements worldwide, underlining the threat of climate change.

NTD programmes need to better understand the burden and the extent to which these are singular or multiple intersecting challenges (Figure 1) to help to develop bespoke strategies and direct resources appropriately. This is important as some countries will experience more conflict- than climate, others more climate- than conflict-, or a complex combination of both conflict-climate.

Figure 1 here

Figure 1. Intersecting challenges of conflict, climate, and related internal displacement

We conducted a cross-sectional ecologic study to better understand the burden of conflict-, violence- and natural disaster-related internal displacements in NTD endemic countries, and the extent to which these are singular or multiple intersecting challenges, to provide a basis from which bespoke strategies could be developed and resource allocation could be appropriately directed. We focused on countries in the World Health Organization (WHO) African region that are endemic for at least one of the five NTDs requiring preventive chemotherapy as a main intervention: lymphatic filariasis, onchocerciasis, schistosomiasis, soil-transmitted helminths, and trachoma.

For peer review only - http://bmjopen.bmj.com/site/about/guidelines.xhtml

METHODS

Study design

A cross-sectional ecologic study design was used to examine and compare the NTD, population, and internal displacement factors and outcomes across different countries for the 2020-2021 period.

Neglected Tropical Disease and population data sources, classifications, and stratification

The NTD and population data were obtained from the Expanded Special Project for Elimination of NTDs (ESPEN) data portal, which collates data from national programmes and stakeholders on the various activities related to the implementation of MDA and/or morbidity management and disability (MMDP).[24] For each country, we summarised i) the number of NTDs requiring preventive chemotherapy and ii) the estimated country population based on 2020 data.

We then classified the countries endemic with four or five NTDs as '*High NTD number*' to reflect the complexity of managing many diseases and programmes, while the other countries were classified as '*Mod(erate)-Low NTD number*.' Second, we classified the ten countries with the highest populations (as an arbitrary threshold) as *'High population'* and others as '*Mod-Low population*'. Third, we combined the various classifications to develop four distinct categories, as shown here, and allocated them accordingly to each country.

NTD-population categories

- *High NTD number + High population*
- *High NTD number + Mod-Low population*
- *Mod-Low NTD number + High population*
- *Mod-Low NTD number + Mod-Low population*

Internal displacement data sources, classifications, and stratification

The data on internal displacements were obtained from the IDMC data portal, which monitors and collates country-specific data on internal displacements associated with conflict and generalised violence, and natural disasters (weather-related and geophysical) from a variety of sources.[23] Internal displacement is referred to as the total number of involuntary or forced movements that have taken place within the borders of a country within a defined period e.g., between 1st January and 31st December 2021, and aims to provide a comprehensive cumulative figure, which may include people being displaced once or several times.

Conflict displacements are referred to as the total number of internal displacements reported as a result of conflict and/or violence events in a reporting year, and natural disaster displacements as

For peer review only - http://bmjopen.bmj.com/site/about/guidelines.xhtml

the total number of internal displacements reported as a result of natural disasters in a reporting year. For this paper focusing on the concept of climate, the related internal displacements are referred to as natural disasters internal displacements as defined by IDMC and only include countries with reported data for 2021.

For each country, we summarised the number of conflict-and natural disaster- internal displacements based on 2021 reported data[23] and calculated crude rates of internal displacement per 100,000 population based on ESPEN population data.[24] Countries with no conflict and climate reported data were not included in the analysis.

We then classified the 10 countries with the highest number and the highest rates of conflict-or disaster internal displacement as '*High conflict*' or '*High disaster*' and others as '*Mod-Low conflict*' or '*Mod-Low disaster*'. Second, the conflict and natural disaster classifications were then combined to develop four distinct categories each for numbers and rates as shown here and allocated to each country.

Displacement numbers and rate categories

- *High conflict + High disaster*
- *High conflict + Mod-Low disaster*
- *Mod-Low conflict + High disaster*
- *Mod-Low conflict + Mod-Low disaster*

Combined categories

All NTD, population and internal displacement categories were then combined to determine the overall patterns, and to help identify countries with high risk or vulnerability. For this paper, we highlighted selected high-risk combinations, which included the countries with a *High NTD number + High population* and a *High NTD number + Mod-Low population.*

Drivers of natural disaster internal displacement

To better understand the drivers of natural disaster internal displacement, we summarised the available IDMC data on hazard types in the countries with the highest natural disaster internal displacement numbers and rates in 2021. Hazard types were categorised as climate or weather-related (drought, flood, extreme temperature, storm, wet mass movement, and wildfire) and geophysical (earthquake, volcanic eruption, dry mass movement). The equivalent data for conflict internal displacement in 2021 were not available from this source and therefore not included in this paper.

For peer review only - http://bmjopen.bmj.com/site/about/guidelines.xhtml

Data management and mapping

The NTD, population, and internal displacement data and related classifications and risk categories for each country were compiled into a Microsoft Excel file for descriptive analysis and mapping. Maps for all classifications and stratifications were created using mapping software ArcGIS 10.8.1 (ESRI, Redland CA) and national administrative boundaries from the ESPEN data portal.[24]. First, NTD, population, and international displacement individual classifications (e.g., *High, Mod-Low*) were mapped. Second, the different categories (e.g., *High+High, High+Mod-Low, Low+Low*) of the different factors were mapped to highlight the variety of risk combinations. Finally, the countries with high-risk combinations for all factors (e.g., *High+High+High+High)* were mapped to highlight the countries with high risk or vulnerability.

Data limitations

We acknowledge that the use of open access data sources may have limitations if they are incomplete, and that our focus on a few selected data sources may cause unforeseen biases.

Patient and public involvement

No patient and/or members of the public were included in this study.

RESULTS

In total, 45 countries in the WHO African region had data on NTDs and populations (excluding Mauritius, Seychelles), and 32 countries reported data on conflict and/or natural disaster internal displacements; 16 countries reported both conflict and natural disaster; 15 countries reported natural disaster and one country reported conflict only internal displacement for 2021.

Neglected Tropical Diseases and population at risk

The total population in the 45 countries with data on NTDs was 1.1 billion. Thirty-one countries had a high number of NTDs: 12 countries endemic with five NTDs and 19 countries endemic with four NTDs (Figure 2A). The 10 countries with the highest populations are shown in Figure 2B, and of these, eight had both *High NTD number + High population* (Figure 2C) including (in descending order): Nigeria (est. 197.4M), Democratic Republic of Congo (DRC; 105.7M), Ethiopia (105.7M), Tanzania (mainland and Zanzibar - 55.9M), Kenya (52.0M), Uganda (41.0M), Angola (31.6M), Mozambique (29.9M) – totalling more than 619M people (Table 1).

For peer review only - http://bmjopen.bmj.com/site/about/guidelines.xhtml

Twelve countries had *Mod-Low NTD number + Mod-Low population* (Botswana, Cape Verde, Comoros, Eritrea, Eswatini, Lesotho, Mauritania, Namibia, Rwanda, Sao Tome and Principe, The Gambia, Togo), while 24 countries had a combination of *High NTD number + Mod-Low population* and one country (South Africa) had a *Mod-Low NTD number + High population* (Figure 2C).

Figure 2 here

Figure 2. Summary maps of Neglected Tropical Diseases, populations, and internal displacement numbers, rates classifications and risk combinations

Conflict and natural disaster internal displacement numbers and rates

Numbers: The total number of internal displacements in 2021 in the 31 high NTD endemic countries was 2.2M. The conflict displacement classifications for each country are shown in Figure 2D; the *High conflict* displacement numbers ranged from 0.1M-5.1M across 10 countries: Ethiopia (5.14M), DRC (2.71M), Burkina Faso (0.68M), Central African Republic (CAR; 0.50M), South Sudan (0.43M), Mali (0.25M), Nigeria (0.38M), Mozambique (0.19M), Cameroon (0.13M) and Niger (0.11M); the *Mod-Low Conflict* numbers across seven countries ranged from 0.94M – 0.04M.

The natural disaster displacement classifications are shown in Figure 2E; the *High Disaster* numbers ranged from 0.24M-0.89M across 10 countries, reported in DRC (0.89M), South Sudan (0.51M), Ethiopia (0.24M), Niger (0.12M), Burundi (0.09M), Tanzania (0.05M), Uganda (0.05M), Mozambique (0.04M), Kenya (0.04M), Nigeria (0.02M); the *Mod-Low disaster* numbers in 20 countries ranged from 255 – 0.02M.

Six countries had both *High conflict + High disaster* internal displacement numbers, including (in descending order) Ethiopia (5.38M combined), DRC (3.60M), South Sudan (0.93M), Nigeria (0.40M), Mozambique (0.23M) and Niger (0.23M) (Figure 2F) – totalling more than 10.8M conflict and natural disaster combined internal displacements in 2021 (Table 1). A summary of the overall internal displacement numbers in the selected high NTD endemic countries is shown in Figure 3A, and highlights the descending order and differences between countries with *High populations* and *Mod-low populations*.

Table 1 here

For peer review only - http://bmjopen.bmj.com/site/about/guidelines.xhtml
For peer review only

Rates: The conflict internal displacement classifications are shown in Figure 2G; the *High conflict* displacement rates (Table 1) ranged from 252.7 – 9020.3 per 100,000 population, reported in CAR (9020.3 per 100,000), Ethiopia (4867.1), South Sudan (3255.6), Burkina Faso (3176.4), DRC (2564.7), Mali (1187.2), Mozambique (625.3), Cameroon (504.0), Niger (475.1), Chad (252.7); the *Mod-Low conflict* displacement rates ranged from 0.082 - 190.2 per 100,000.

The natural disaster internal displacement classifications are shown in Figure 2H; the *High disaster* displacement rates (Table 1) ranged from 144.1-3832.6 per 100,000 population, reported in South Sudan (3832.6 per 100,000), DRC (839.8), Burundi (754.7), Niger (507.6), CAR (429.3), Sao Tome and Principe (237.8), Ethiopia (227.2), Namibia (165.0), Mozambique (145.5), Chad (144.1); the *Mod-Low disaster* displacement rates ranged from 3.2 - 121.4 per 100,000.

Seven countries had both *High conflict + High disaster* internal displacement rates, including (in descending order) CAR (9449.7 combined), South Sudan (7088.1), Ethiopia (5094.3), DRC (3404.5), Niger (982.7), Mozambique (770.8) and Chad (398.8) (Figure 2I). A summary of the overall internal displacement rates per 100,000 in the selected high NTD endemic countries is shown in Figure 3B, and highlights the descending order and differences between countries with *High populations* and *Mod-low populations*.

.

For peer review only

Table 1. Summary of populations, and internal displacement data and high-risk combinations in selected countries with high number of Neglected Tropical Diseases

Location‡	Population *	Internal displacement numbers†			Internal displacement rates per 100,000 population			Combined classifications
Country	Population	Conflict No.	Natural Disaster No.	Conflict Disaster No.	Conflict rate	Natural Disaster rate	Conflict Disaster rate	NTD, Population Conflict, Natural Disaster
**High NTD number + High population**								
DRC	105.7M	2,711,783	888,000	3.60M	2,564.7	839.8	3,404.5	High NTD-population+ conflict-disaster
Ethiopia	105.7M	5,142,356	240,009	5.38M	4867.1	227.2	5,094.3	High NTD-population+ conflict-disaster
Mozambique	29.9M	187,057	43,512	0.23M	625.3	145.5	770.8	High NTD-population+ conflict-disaster
Nigeria	197.4M	375,552	24,366	0.40M	190.2	12.3	202.6	
Tanzania	55.9M	0	46,707	0.05M	0	85.6	85.6	
Kenya	52.0M	4,634	36,442	0.04M	8.9	70.1	79.0	
Uganda	41.0M	1,278	46,690	0.05M	3.1	113.8	116.9	
Angola	31.6M	0	21727	0.02M	0	68.8	68.8	
**Summary**	**619M+**	**8,422,660**	**1,347,453**	**9.77M**	**0-4,867.1**	**12.3-839.8**	**68.8 – 5,094.3**	
**High NTD number + Mod-Low population**								
South Sudan	13.2M	429,432	505,544	0.93M	3255.6	3,832.6	7,088.1	High NTD-conflict-disaster
Niger	23.2M	110,203	117,747	0.23M	475.1	507.6	982.7	High NTD-conflict-disaster
Burkina Faso	21.5M	682,245	0	0.68M	3176.4	0	3176.4	High NTD - conflict
Cameroon	26.1M	131,452	1,764	0.13M	504.0	6.8	510.8	High NTD– conflict
CAR	5.5M	495,600	23,589	0.52M	9,020.3	429.3	9,449.7	High NTD - conflict
Mali	21.0M	249,174	5,994	0.26M	1,187.2	28.6	1,215.7	High NTD - conflict
Burundi	11.5M	94	86,885	0.09M	0.8	754.7	755.5	High NTD - disaster
Chad	16.8M	42,355	24,144	0.07M	252.7	144.1	398.8	
**Summary**	**138.8M**	**2,140,555**	**765,667**	**2.91M**	**0.8-9,020.3**	**0 – 3,832.6**	**398.8 – 9,449.7**	

‡ All countries have a high number of NTDs
*ESPEN data 2021[24]
† IDMC data 2019-2021[23]
Shading represents countries with a classified High NTD number, High population, or *High Conflict* and/or *High Natural disaster* internal displacement numbers and/or rates

Figure 3 here

Figure 3. Summary of overall internal displacement numbers and rates in selected countries with high numbers of Neglected Tropical Diseases

Risk combinations

Of the countries with *High NTD number + High population*, we found that DRC, Ethiopia, Mozambique, and Nigeria had *High conflict + High disaster* displacement numbers (Figure 2J), and DRC, Ethiopia, and Mozambique had *High conflict + High disaster* displacement rates (Figure 2K) in 2021. Collectively, these three countries reported more than 241M people at risk of four to five NTDs and 9.2M conflict and/or natural disaster internal displacements in 2021 alone; these may be considered the most at risk or vulnerable countries in the African region. Nigeria was found to have *High conflict + High disaster* displacement numbers, while Tanzania, Kenya, and Uganda had among the highest *High disaster* displacement numbers (Table 1).

Of the countries with high NTD numbers and displacement, we found South Sudan and Niger to have both *High conflict + High disaster* number and rates, while Burkina Faso, Cameroon, CAR, and Mali had both *High conflict* numbers and rates, and Burundi had both *High disaster* displacement numbers and rates in 2021 (Table 1).

Drivers of natural disaster internal displacement

The number of reported natural disaster hazard events in 2021 varied across countries, with the highest frequency reported in Burundi (n=80), Uganda (n=60) and South Sudan (n=31), and the highest number of hazard-related internal displacements reported in DRC (0.89M), South Sudan (0.51M), and Ethiopia (0.24M) (Table 2). In all countries, weather-related hazards were most common with floods the dominant hazard type, accounting for around half (55.2%) of all natural disaster displacements across 12 countries, except DRC where a volcanic disruption led to 0.6M displacements, and in Mozambique where a storm led to 0.04M displacements. In total, 150 flood events across 11 countries caused 0.85M natural disaster hazard related internal displacements. Other causes of internal displacement included mass wet or dry movement and wildfires but affected far fewer numbers. A summary of the overall number and frequency of natural disaster internal displacements by hazard type in the selected high NTD endemic countries is shown in Figure 4 A and B, and highlights the predominance of floods.

For peer review only - http://bmjopen.bmj.com/site/about/guidelines.xhtml

Table 2. Summary of natural disaster internal displacements by hazard type in selected countries with high number of Neglected Tropical Diseases

Country	Total Population.	Total number (No.) of Natural Disaster Displacements	Total rate of Natural Disaster Displacements	Total No. hazard events	No. of natural disaster displacements by hazard type (No. of hazard events)			
					Flood	Drought	Storm	Other*
**High NTD number, High population, High climate numbers or rates**								
DRC	105.7M	0.89M	2564.7	21	0.26M (n=10)		0.002M (n=8)	0.6M (n=3)
Ethiopia	105.7M	0.24M	839.8	7	0.19M (n=4)	0.05M (n=1)		0.00001 (n=2)
Mozambique	29.9M	0.04M	145.5	2			0.04M (n=2)	
Nigeria	197.4M	0.02M	12.3	31	0.02M (n=31)			
Tanzania	55.0M	0.05M	85.6	6	0.02M (n=2)		0.03M (n=4)	
Kenya	52.0M	0.04M	70.1	3	0.04M (n=2)		0.000M (n-1)	
Uganda	41.0M	0.05M	113.8	60	0.04M (n=24)		0.005M (n=28)	0.001M (n=8)
**Summary**	**586.7M**	**1.33**	**12.3 – 2564.7**	**130**	**0.57M (n=73)**	**0.05M (n=1)**	**0.08 (n=43)**	**0.60 (n=13)**
**High NTD number, Mod-Low population, high natural disaster numbers or rates**								
South Sudan	13.2M	0.05M	3832.6	39	0.05M (n=39)			
Niger	23.2M	0.12M	507.6	1	0.12M (n=1)			
CAR	5.5M	0.02M	429.3	16	0.02 (n=16)			
Burundi	11.5M	0.26M	754.7	79	0.07M (n=15)		0.013M (n=63)	0.0002 (n=1)
Chad	16.8M	0.02M	144.1	6	0.02 (n=6)			
**Summary**	**70.2M**	**0.47M**	**144.1 – 3832.6**	**141**	**0.28M (n=77)**		**0.013M (n=63)**	**0.0002 (n=1)**

Data source IDMC (numbers rounded)[23]
*Other category included – wet or dry mass movement and/or wildfires

For peer review only

Figure 4 here

Figure 4. Proportion of overall natural disaster internal displacement numbers and frequency of events by hazard type

DISCUSSION

This paper highlights that many African countries have high numbers of NTDs, which require a great deal of coordination and logistics in-country to implement routine NTD programming.[24] In the eight most populous endemic countries, we quantified that more than half a billion people will need multiple NTD interventions and long-term surveillance activities in the next decade. Routine NTD programming has been and will be negatively impacted by large population displacements, which adds to the existing difficulties of determining accurate population numbers in countries where there is a lack of regular census data.[25,26] The ESPEN data portal provides population estimates[24] and could start to collate data on internal displacements from various sources, including NTD programme managers who are experiencing the challenges first hand and may provide more detailed information and invaluable insights into these problems.

Alarmingly, here we show in just one year that Ethiopia, DRC, South Sudan, Nigeria, Mozambique, and Niger reported more than 10M internal conflict and natural disaster internal displacements and have among them the highest conflict and natural disaster rates per population. If current trends continue or increase, these countries are at high risk of not reaching the NTD road map targets and will require significant technical and financial support from stakeholders if global goals are to be met.[1] This is critical as these six countries have among the lowest levels of human development in the world,[27] and may not be able to access and mobilise in-country resources needed to accelerate programmatic action, intensify cross-cutting approaches, and change operating models to facilitate enhanced country ownership as set out in the NTD road map 2021-2030.[1]

We limited our analysis to one internal displacement data source but advocate that future research should aim to triangulate information from other data sources to build a comprehensive picture of the situation. We also acknowledge that the use of open-source data may have limitations if they are incomplete or may be updated or changed over time, and that the analyses may only be as good as the available data allow them to be at the time of access. For example, there are frequently issues with the accuracy of the NTD and population data, and additional data on internal displacements for

For peer review only - http://bmjopen.bmj.com/site/about/guidelines.xhtml

this study may now be available. Therefore, the analyses and outcomes presented in this paper are indicative of the scale of the problem, and numbers should not be taken as being precise.

A further limitation was that we applied simple classifications and risk combinations to the NTD, population, and internal displacement figures to allow us to initially focus on countries most affected as a means of understanding the magnitude of these complex challenges. This does not imply that countries with lower numbers or rates of NTDs, population, conflict and natural disaster internal displacements are less of a priority for NTD programmes, but rather the magnitude is different. All endemic countries in Africa and elsewhere will need support to address these challenges and future work should consider more advanced analysis applied at a subnational level and include additional compounding factors such as health infrastructure, community accessibility, and strength of the NTD programme.[15,20]

Further, future work also needs to consider the spatial-temporal patterns and geographical overlap of conflict and natural disaster driven displacement to identify hotspots, and determine the long-term implications and strategies needed for NTD programme activities. For example, if there is protracted conflict in an area, then programmes can adapt their methods of programmatic delivery, target non-conflict areas, or identify methods of treating displaced populations that have sought refuge in secure areas.[17] If flooding is increasingly predictable in an area, then programmes can adapt the timing of programme activities in that area or identify alternate treatment sites for those displaced by the flooding.

The African continent is predicted to continue experiencing negative effects from climate change due to increasing temperatures, rising sea-levels, cumulative failed rainy seasons, drought, and shrinking surface water.[8] These events and resulting food and water scarcity can feed into political instability, and exacerbate ongoing tensions thereby creating a vulnerability multiplier for those at risk of conflict, natural disasters, and NTDs. Climate change is an escalating security threat,[28–31] and an understanding of the key drivers is critical in Africa and elsewhere. Here we highlight that most natural disaster hazards were climate or weather-related events, especially flooding, which led to nearly one million displacements in 11 African countries in one year alone. However, the impact of these events on disease transmission dynamics in highly endemic areas, and how they affect programmes, is largely unknown as very little research has been conducted in Africa and elsewhere.[4–6]

For peer review only - http://bmjopen.bmj.com/site/about/guidelines.xhtml

Moving forward NTD programmes will need to engage a range of other stakeholders that are working towards addressing and/or mitigating the impact of conflict and/or climate change at an international and national level. [32] This will help to identify country specific needs and create opportunities for cross-sector partnerships, good coordination, and the potential co-production and co-implementation of effective strategies.

Recommendations

We advocate for a "call to action" to encourage policy makers, programme managers, researchers, implementing partners and donors to develop, implement, and evaluate a set of strategies to better assess NTD burden in areas at risk or experiencing conflict and climate related natural disasters, and deliver interventions that are urgently required. Here we focus on African countries, but the same methodologies may be applied to different WHO regions to understand the distinct and common challenges. There is a need to build on core principles and adapted survey strategies already being developed and implemented by experienced teams that may address these special populations and problems.[14–16,33,34] It will also be important to learn from countries that have implemented efficient and timely responses during conflicts and disasters to internally displaced populations through cross-sectoral collaboration.

Though national capacity to respond to humanitarian emergencies can be limited, as a first step we suggest an overarching set of recommendations that include the development of preferred practices; data platform; decision support tool, advocacy and communication and implementation research strategies as outlined in Box 1.

For peer review only - http://bmjopen.bmj.com/site/about/guidelines.xhtml

Box 1. Overarching recommendations to address complex challenges in NTD endemic countries

- PREFERRED PRACTICES: A set of informed preferred practice documents outlining potential step-by step processes and methods to address singular or multiple intersecting conflict- climate-displacement challenges that impede NTD programmatic activities.
- DATA PLATFORM: A central data portal that combines data for each country on: NTD burden, population size, internal and cross-border displacement numbers (by conflict, natural disaster, and hazard type categorisation) and administrative boundaries.
- DECISION SUPPORT TOOL: A practical online tool that facilitates the national NTD programme teams to input and analysis conflict-climate-displacement data to assess risk and support programmatic decision-making at implementation unit or district level.
- ADVOCACY AND COMMUNICATION. An advocacy and communication plan with goals to document and inform national and international stakeholders of the key threats and success stories related to the conflict-climate-displacement challenges that NTD programmes confront.
- IMPLEMENTATION RESEARCH: A strategic research agenda outlining the range of conflict- climate-displacement challenges, and the potential research questions, methodological approaches, and funding opportunities that could be drawn on by NTD programmes and academic partners.

CONCLUSION

This paper highlights the urgent need for health policies and guidelines to address the escalating risks of conflict, climate change-induced natural disasters, and associated internal displacement of populations in NTD endemic countries in Africa. We have shown that publicly available data sources could potentially be used by national policy makers and NTD programmes as a first step to better understanding the intersecting challenges and their level of risk and vulnerability. This would allow them to develop risk assessments and mitigation strategies that may be tailored to the local context and health needs of internally displaced persons, given that the types and burden of NTDs, and the causes of internal displacement, vary within and between countries. There is a need to build new stakeholder collaborations and a collective agenda for these neglected conflict-climate-displacement challenges, so that NTD programmes can meet the 2021-2030 road map targets and 2030 Agenda for Sustainable Development and ensure that no one is left behind.

For peer review only - http://bmjopen.bmj.com/site/about/guidelines.xhtml

CONTRIBUTORS

LAK-H contributed to the conception, methodology, formal analysis, writing-original draft. EH-E, and AMS contributed to the interpretation, writing of the recommendations, writing review and editing. JW and FA contributed to the interpretation, writing review and editing. All authors approved the final version. All authors are members of the Neglected Tropical Diseases Non-Governmental Organisation Network (NNN), Conflict and Humanitarian Emergency (C&HE) Cross-Cutting Group - Mapping Task Team https://www.ntd-ngonetwork.org/cross-cutting-groups/conflict-and-humanitarian-emergencies.

DATA AVAILABILITY STATEMENT

All data relevant to this study are included in the article or available in the public, open access repositories.

ETHICS STATEMENT

Patient consent for publication

This study does not involve human participants and ethical approval was not required.

COMPETING INTERESTS

None declared

FUNDING

The work was not funded by a specific grant. The University of Liverpool provided funding for the open access publishing cost.

For peer review only

For peer review only - http://bmjopen.bmj.com/site/about/guidelines.xhtml

REFERENCES

1 World Health Organization. Ending the neglect to attain the sustainable development goals: a road map for neglected tropical diseases 2021–2030. Geneva: World Health Organization, 2020 https://apps.who.int/iris/handle/10665/338565 (accessed Aug 4, 2022).

2 Bangert M, Molyneux DH, Lindsay SW, Fitzpatrick C, Engels D. The cross-cutting contribution of the end of neglected tropical diseases to the sustainable development goals. *Infectious Diseases of Poverty* 2017; **6**: 73.

3 United Nations.Department of Economic and Social Affairs. Sustainable Development Goals. Sustainable Development. https://sdgs.un.org/goals (accessed Dec 14, 2022).

4 Tidman R, Abela-Ridder B, de Castañeda RR. The impact of climate change on neglected tropical diseases: a systematic review. *Transactions of The Royal Society of Tropical Medicine and Hygiene* 2021; **115**: 147–68.

5 Bryson JM, Bishop-Williams KE, Berrang-Ford L, *et al.* Neglected Tropical Diseases in the Context of Climate Change in East Africa: A Systematic Scoping Review. *The American Journal of Tropical Medicine and Hygiene* 2020; **102**: 1443–54.

6 Booth M. Chapter Three - Climate Change and the Neglected Tropical Diseases. In: Rollinson D, Stothard JR, eds. Advances in Parasitology. Academic Press, 2018: 39–126.

7 Eckstein D, Künzel V, Schäfer L. Global Climate Risk Index 2021 Who Suffers Most Extreme Weather Events? Weather-Related Loss Events in 2019 and 2000-2019. 2021.

8 World Meteorological Organization. State of Climate Africa 2021. 2022 https://library.wmo.int/index.php?lvl=notice_display&id=22125#.Y5l2B-zP2lM (accessed Dec 14, 2022).

9 Zielinski C. COP27 climate change conference: urgent action needed for Africa and the world. *BMJ Global Health* 2022; **7**: e010965.

10 Palik J, Rustad SA, Methi F. Conflict Trends in Africa, 1989–2019. .

11 Raleigh C, Linke A, Hegre H, Karlsen J. Introducing ACLED: An Armed Conflict Location and Event Dataset: Special Data Feature. *Journal of Peace Research* 2010; **47**: 651–60.

12 Yaya G. Lessons learned in the implementation of programmes to eliminate trachoma within conflict zones. *Trans R Soc Trop Med Hyg* 2022; : trac061.

13 Du RY, Stanaway JD, Hotez PJ. Could violent conflict derail the London Declaration on NTDs? *PLoS Negl Trop Dis* 2018; **12**: e0006136.

14 McPherson, Scott. Working outside our comfort zone: Fighting NTDs in conflict areas. Working outside our comfort zone: Fighting NTDs in conflict areas. https://iamtropmed.org/blog/2019/11/21/working-outside-our-comfort-zone-fighting-ntds-in-conflict-areas (accessed March 19, 2023).

15 Harvey D, Shu'aibu J, Debam MT, Aba AK, Torres-Vitolas CA. How can the neglected tropical disease community be inclusive and equitable in programme delivery? Reaching refugees and internally displaced persons through integrating a 'leave no one behind' approach. *Int Health* 2022; **14**: ii33–7.

For peer review only

For peer review only - http://bmjopen.bmj.com/site/about/guidelines.xhtml

16 Neglected Tropical Disease NGO Network (NNN). Conflict and Humanitarian Emergencies. https://www.ntd-ngonetwork.org/cross-cutting-groups/conflict-and-humanitarian-emergencies (accessed Aug 24, 2022).

17 Kelly-Hope LA, Sanders AM, Harding-Esch E, *et al.* Complex emergencies and the control and elimination of neglected tropical diseases in Africa: developing a practical approach for implementing safe and effective mapping and intervention strategies. *Confl Health* 2021; **15**: 18.

18 Kelly-Hope LA, Molyneux DH. Quantifying conflict zones as a challenge to certification of Guinea worm eradication in Africa: a new analytical approach. *BMJ Open* 2021; **11**: e049732.

19 Cantor D, Swartz J, Roberts B, *et al.* Understanding the health needs of internally displaced persons: A scoping review. *J Migr Health* 2021; **4**: 100071.

20 Adams MW, Sutherland EG, Eckert EL, Saalim K, Reithinger R. Leaving no one behind: targeting mobile and migrant populations with health interventions for disease elimination—a descriptive systematic review. *BMC Med* 2022; **20**: 172.

21 World Health Organization. Summary of global update on implementation of preventive chemotherapy against NTDs in 2020. 2021.

22 Bockarie MJ, Kelly-Hope LA, Rebollo M, Molyneux DH. Preventive chemotherapy as a strategy for elimination of neglected tropical parasitic diseases: endgame challenges. *Philos Trans R Soc Lond B Biol Sci* 2013; **368**: 20120144.

23 Internal Displacement Monitoring Centre (IDMC). Global Internal Displacement Database. Data (2022). https://www.internal-displacement.org/database/displacement-data. (accessed Aug 9, 2022).

24 World Health Organization. Expanded Special Project for Elimination of Neglected Tropical Diseases (ESPEN). 2022. https://espen.afro.who.int/ (accessed Aug 9, 2022).

25 Carlin, Alexandra. Why we should reconsider population data for disease control and public health programmes. BMC BugBitten. 2021; published online Nov 26. https://blogs.biomedcentral.com/bugbitten/2021/11/26/population-data-disease-control-public-health/ (accessed Oct 10, 2022).

26 Ratnayake R, Abdelmagid N, Dooley C. What we do know (and could know) about estimating population sizes of internally displaced people. *Journal of Migration and Health* 2022; **6**: 100120.

27 United Nations Develop Programme (UNDP). Human Development Report 2021-22: Uncertain Times, Unsettled Lives: Shaping our Future in a Transforming World. New York, 2022 https://hdr.undp.org/data-center/human-development-index#/indicies/HDI (accessed Oct 10, 2022).

28 United Nations Environment Programme (UNEP). Climate change and security risks. Disasters and conflicts. 2022. https://www.unep.org/explore-topics/disasters-conflicts/what-we-do/disaster-risk-reduction/climate-change-and-security (accessed Oct 13, 2022).

29 Brown O, Hammill A, Mcleman R. Climate change as the 'new' security threat: implications for Africa. *International Affairs* 2007; **83**: 1141–54.

For peer review only

For peer review only - http://bmjopen.bmj.com/site/about/guidelines.xhtml

30 Evans MS, Munslow B. Climate change, health, and conflict in Africa's arc of instability. *Perspect Public Health* 2021; **141**: 338–41.

31 Mach KJ, Kraan CM, Adger WN, *et al.* Climate as a risk factor for armed conflict. *Nature* 2019; **571**: 193–7.

32 United Nations Office for the Coordination of Humanitarian Affairs (OCHA). Humanitarian System/Clusters/What is the Cluster approach? https://www.humanitarianresponse.info/en/coordination/clusters/what-cluster-approach (accessed March 19, 2023).

33 Sanders AM, Abdalla Z, Elshafie BE, *et al.* Prevalence of trachoma within refugee camps serving South Sudanese refugees in White Nile State, Sudan: Results from population-based surveys. *PLoS Negl Trop Dis* 2019; **13**: e0007491.

34 The International, Coalition for Trachoma Control, (ICTC). THE END IN SIGHT: From collaboration to elimination, strategic plan 2022–2030. 2022; published online May. https://www.trachomacoalition.org/sites/default/files/content/resources/files/ICTC%20Strategic%20Plan.pdf (accessed Oct 10, 2022).

For peer review only

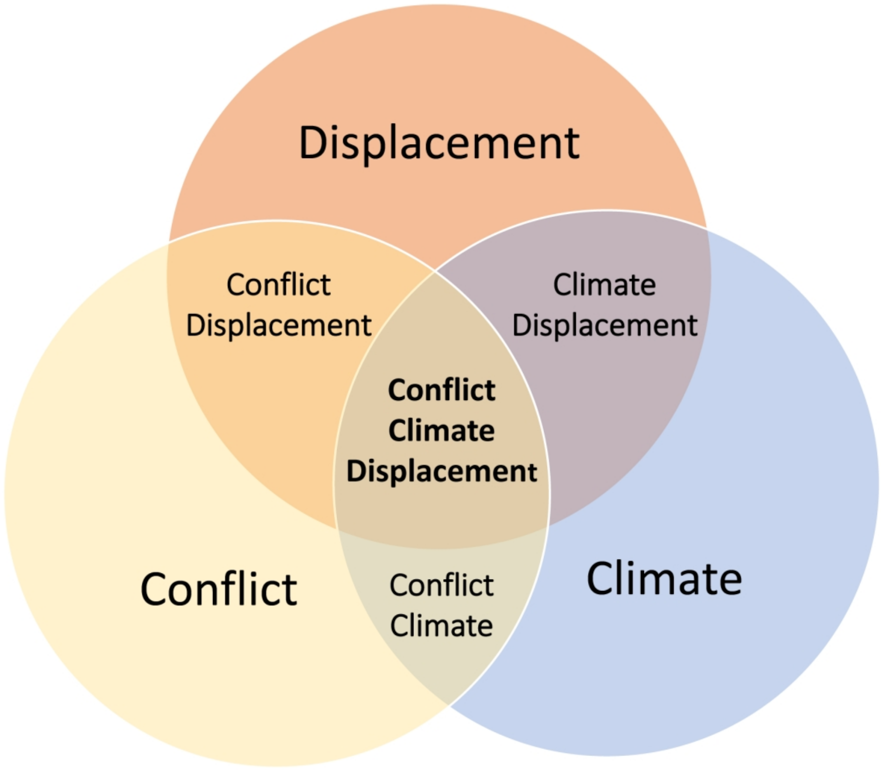

164x147mm (300 x 300 DPI)

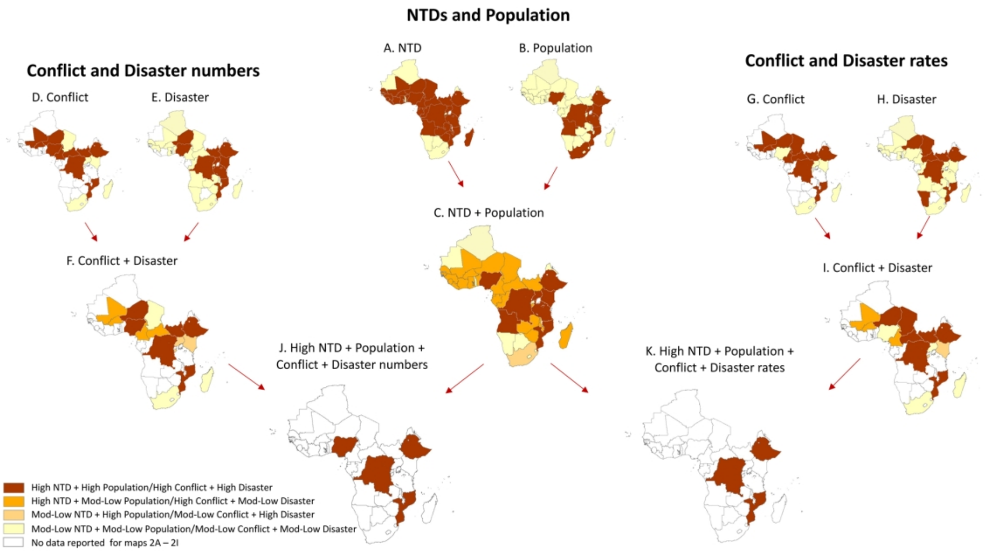

Figure 2

170x94mm (300 x 300 DPI)

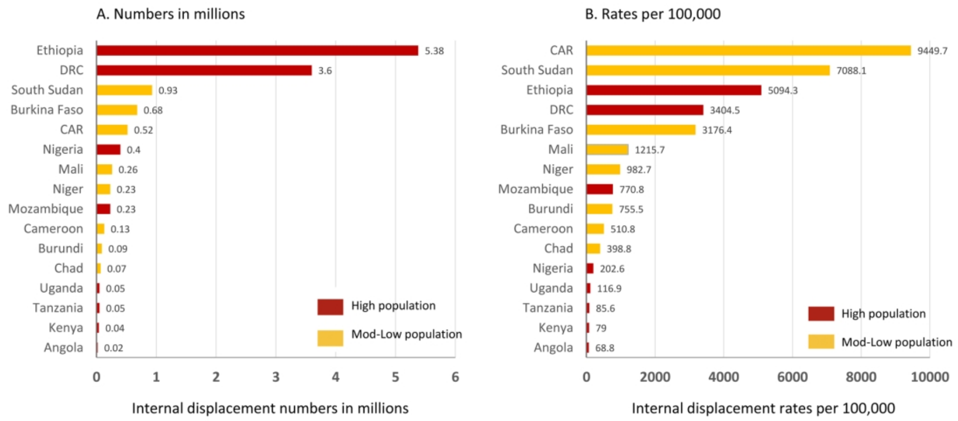

Figure 3

170x74mm (300 x 300 DPI)

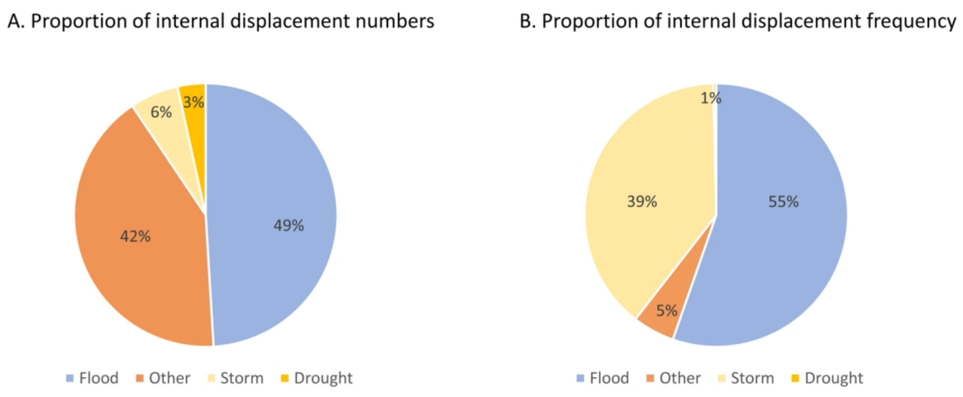

Figure 4

150x64mm (300 x 300 DPI)

STROBE Statement—Checklist of items that should be included in reports of ***cross-sectional studies***

	Item No	Recommendation	Page No
**Title and abstract**	1	(*a*) Indicate the study's design with a commonly used term in the title or the abstract	2
		(*b*) Provide in the abstract an informative and balanced summary of what was done and what was found	2
**2Introduction**			
Background/rationale	2	Explain the scientific background and rationale for the investigation being reported	4-5
Objectives	3	State specific objectives, including any prespecified hypotheses	5
**Methods**			
Study design	4	Present key elements of study design early in the paper	5
Setting	5	Describe the setting, locations, and relevant dates, including periods of recruitment, exposure, follow-up, and data collection	6
Participants	6	(*a*) Give the eligibility criteria, and the sources and methods of selection of participants	6-7
Variables	7	Clearly define all outcomes, exposures, predictors, potential confounders, and effect modifiers. Give diagnostic criteria, if applicable	6-7
Data sources/ measurement	8*	For each variable of interest, give sources of data and details of methods of assessment (measurement). Describe comparability of assessment methods if there is more than one group	*6-7*
Bias	9	Describe any efforts to address potential sources of bias	6
Study size	10	Explain how the study size was arrived at	6-7
Quantitative variables	11	Explain how quantitative variables were handled in the analyses. If applicable, describe which groupings were chosen and why	6-7
Statistical methods	12	(*a*) Describe all statistical methods, including those used to control for confounding	N/A
		(*b*) Describe any methods used to examine subgroups and interactions	6-7
		(*c*) Explain how missing data were addressed	6
		(*d*) If applicable, describe analytical methods taking account of sampling strategy	6-7
		(*e*) Describe any sensitivity analyses	N/A
**Results**			
Participants	13*	(a) Report numbers of individuals at each stage of study—eg numbers potentially eligible, examined for eligibility, confirmed eligible, included in the study, completing follow-up, and analysed	N/A
		(b) Give reasons for non-participation at each stage	N/A
		(c) Consider use of a flow diagram	Figure 2
Descriptive data	14*	(a) Give characteristics of study participants (eg demographic, clinical, social) and information on exposures and potential confounders	8-12
		(b) Indicate number of participants with missing data for each variable of interest	N/A
Outcome data	15*	Report numbers of outcome events or summary measures	8-12

For peer review only - http://bmjopen.bmj.com/site/about/guidelines.xhtml

Main results	16	(*a*) Give unadjusted estimates and, if applicable, confounder-adjusted estimates and their precision (eg, 95% confidence interval). Make clear which confounders were adjusted for and why they were included	N/A
		(*b*) Report category boundaries when continuous variables were categorized	8-12
		(*c*) If relevant, consider translating estimates of relative risk into absolute risk for a meaningful time period	N/A
Other analyses	17	Report other analyses done—eg analyses of subgroups and interactions, and sensitivity analyses	8-12
**Discussion**			
Key results	18	Summarise key results with reference to study objectives	13
Limitations	19	Discuss limitations of the study, taking into account sources of potential bias or imprecision. Discuss both direction and magnitude of any potential bias	13-14
Interpretation	20	Give a cautious overall interpretation of results considering objectives, limitations, multiplicity of analyses, results from similar studies, and other relevant evidence	13-14
Generalisability	21	Discuss the generalisability (external validity) of the study results	13-14
**Other information**			
Funding	22	Give the source of funding and the role of the funders for the present study and, if applicable, for the original study on which the present article is based	16

*Give information separately for exposed and unexposed groups.

Note: An Explanation and Elaboration article discusses each checklist item and gives methodological background and published examples of transparent reporting. The STROBE checklist is best used in conjunction with this article (freely available on the Web sites of PLoS Medicine at http://www.plosmedicine.org/, Annals of Internal Medicine at http://www.annals.org/, and Epidemiology at http://www.epidem.com/). Information on the STROBE Initiative is available at www.strobe-statement.org.

For peer review only
